# CD32B1, a versatile non-signaling antibody-binding scaffold for enhanced T cell adhesion to tumor stromal cognate antigens

**DOI:** 10.3389/fimmu.2025.1398757

**Published:** 2025-02-10

**Authors:** Sara W. Feigelson, Tali Dadosh, Nehora Levi, Anita Sapoznikov, Hadas Weinstein-Marom, Dayana Blokon-Kogan, Yahel Avraham, Tamar Unger, Gideon Gross, Rony Dahan, Ronen Alon

**Affiliations:** ^1^ Department of Immunology and Regenerative Biology, Weizmann Institute of Science, Rehovot, Israel; ^2^ Department of Chemical Research Support, Weizmann Institute of Science, Rehovot, Israel; ^3^ Laboratory of Immunology, MIGAL-Galilee Research Institute, Kiryat Shmona, Israel; ^4^ Department of Biotechnology, Tel-Hai College, Upper Galilee, Israel; ^5^ Department of Systems Immunology, Weizmann Institute of Science, Rehovot, Israel; ^6^ Department of Life Sciences Core Facilities, Weizmann Institute of Science, Rehovot, Israel

**Keywords:** cancer immunotherapy, tumor microenvironment, cytotoxic lymphocytes, adoptive cell therapy, Fc receptors, microvilli

## Abstract

Targeting cytotoxic T lymphocytes (CTLs), as chimeric antigen T cells (CAR-T), T cell receptor-engineered (TCR)-T cells or adoptive cell transfer of tumor infiltrating T cells (TILs) to solid tumors is a major therapeutic challenge. We describe a new strategy to confer these lymphocytes with *de novo* adhesiveness to surface proteins enriched in the tumor microenvironment. This approach is based on decorating CTLs with monoclonal antibodies (mAbs) specific to any surface protein of interest within the stroma and the extracelullar matrix of solid tumors. For efficient mAb decoration, we have introduced a mAb binding Fc receptor (FcR) scaffold, FcγRIIB1 (CD32B1), which we found to be enriched on B lymphocyte microvilli (MV). This isoform contains an inhibitory ITIM motif within a cytoplasmic tail anchored to the cortical cytoskeleton. We thus generated a non-signaling CD32B1 mutant lacking the ITIM motif (termed ITIM-less CD32B1, or ILCD32B1) and successfully expressed it in human T cells which normally do not express this FcR. The ILCD32B1 expressing lymphocytes bound multiple IgG1 mAbs whose Fc domain was engineered with a 5-residue substitution to reach a nM range of Fc-FcγCR dissociation constants. The mAb decorated ILCD32B1 expressing T cells could readily adhere to a surface-bound cognate antigen. To broaden the utility of this scaffold, we have also generated a new fusion protein in which the entire Fc binding domain was truncated (tILCD32B1) and replaced with a monomeric streptavidin variant, mSA2, via a CD8 hinge. The molecule, termed mSA2-CD8h-tILCD32B1, was also successfully expressed in T cells, readily and stably bound biotinylated IgG mAbs *in vitro* and once decorated with the biotin labeled mAbs, conferred the T cells with high adhesiveness to multiple surface-coated antigens. mSA2-CD8h-tILCD32B1 expressing human T cells decorated ex vivo with a biotin-labeled mAb retained the antibody for hours after accumulation inside breast tumors implanted in immunodeficient recipient mice. Our results collectively suggest that a non-signaling CD32B1 can be used as a versatile scaffold for mAb decoration of T cells. Our mAb decoration approach can confer new cell adhesive reactivities to improve tumor CTL (CAR-T and TIL) accumulation and retention inside solid tumors.

## Introduction

Therapeutic usage of CTLs such as CAR-T cells at sites of hematopoietic malignancies such as the bone marrow and spleen, or of TILS or TCR-T cells injected into superficial tumors such as melanoma has been highly successful (1–3). Nevertheless, it is still very difficult to efficiently target such CTLs to specific stromal niches within solid primary tumors or metastatic lesions (4). One of the reasons for this poor targeting of therapeutic CTLs is that tumor associated stromal cells express a variety of cell-surface antigens not recognized by effector T cells (5–12). Conferring effector lymphocytes with the capacity to recognize and adhere to tumor enriched surface molecules or to their surrounding extracellular matrix could therefore be a therapeutically promising strategy to improve the accumulation and retention of therapeutic CTLs such as TILs and CAR-T CTLs inside solid tumors.

To achieve this goal, we sought new approaches for the decoration of such CTLs with mAbs specific for any antigens of interest enriched in tumor microenvironments. We reasoned that such surface decorated mAbs should function as surrogates for cell-adhesive receptors but otherwise remain functionally inert, avoiding undesirable signaling into the decorated T cells. Decoration of cells with mAbs can be achieved chemically e.g., by introduction of biotin labeled spacers on membranal proteins and glycolipids but such decoration strategy is difficult to control and is toxic (13). We have therefore developed a new genetic strategy of T cell decoration by introducing into T cells a universal IgG binding scaffold protein composed of the FcR family member FcγRIIB1 (CD32B1). FcγRIIB1 is an alternatively spliced isoform expressed by B lymphocytes (14). This inhibitory ITIM containing FcR contains an extra cytoplasmic tail domain while its shorter alternatively spliced variant FcγRIIB2 is rapidly internalized upon IgG binding (15, 16). The longer tail of FcγRIIB1 anchors this IgG binding receptor to the cortical cytoskeleton and prevents its internalization upon binding of immune complexes and stabilizes it in multimeric assemblies (17–19). CD32B1 has therefore been successfully used as a capping scaffold for various native and Fc engineered mAbs (17–19). As the cytoplasmic tail of CD32B1 contains an immune suppressory ITIM motif, we have eliminated this motif in order to avoid any transmission of inhibitory signals by the IgG occupied FcR. This isoform as well as other CD32B isoforms are not endogenously expressed by T cells (14) and was therefore ectopically expressed in *ex vivo* generated CTLs.

Using super-resolution microscopy, we found that endogenous CD32B1 is located on tips of B cell microvilli while the ectopic FcγR was expressed on the entire T cell surface including microvilli. Since CD32B1 on both B and T lymphocytes binds native IgG1 with moderate affinity, we have used model mAbs whose Fc region was modified with substitutions that increase the affinity of their Fc to CD32B1 by 20-100 fold (20). Once decorated with an Fc-modified mAb, CD32B1 expressing T cells readily adhered to the cognate antigens immobilized on a surface. To further diversify the usage of the CD32B as a mAb scaffold, we have also generated an ITIM-less CD32B1 in which the Fc binding domain was truncated (herein tILCD32B1) and replaced with a monomeric streptavidin variant, mSA2 that binds biotin-labeled proteins at a nM K_D_ range (21–23) and fused via a CD8 hinge. The fusion molecule consisted of a CD8 hinge spacer (termed mSA2-CD8h-tILCD32B1) was also successfully expressed on T cell microvilli and was readily decorated with biotin modified fluorescent probes and with biotin labeled mAbs. As with native CD32B1, T lymphocytes expressing this fusion molecule and decorated with mAbs readily adhered to a model antigen recognized by the decorating mAb. Collectively, our present study indicates that CD32B1 can be used as a multi-functional scaffold for distinct Fc engineered or biotin labeled mAbs with any specificity of interest. The mAb decorated CD32B1 expressing T cells can acquire *de novo* specific recognition of any cell-surface antigen or extracellular matrix component of interest. Our approach could be harnessed for the future targeting of therapeutic CTLs into solid tumors.

## Materials and methods

### Antibodies

Purified (cat.302202) and PE-conjugated (cat.982402) anti-human CD19 (HIB19), purified (cat.303202) and APC-conjugated (cat.303208) anti-human CD32 (FUN-2), purified anti-human IgM (cat.314502, MHM-88), Pacific Blue-conjugated anti-human CD4 (cat.317424, OKT4), APC-conjugated anti-human CD8 (cat.980904, SKI), purified (cat.317301) and FITC-conjugated (cat.317305) anti-human CD3 (OKT3), purified anti-CD28 (cat.302902, CD28.2), purified (cat.304002) and biotin (cat.304004) anti-human CD45 (H130), biotin anti-B220 (cat.103204, RA3-6B2), purified (cat.116102) and biotin (cat.116103) anti-mouse CD54 (YN1/1.7.4), purified (cat.105701) and biotin (cat.105703) anti-mouse CD106 (429(MVCAM.A)), were purchased from Bioleged. Biotinylated recombinant human IgG Fc Avi-tag (cat.AVI110) was purchased from R&D Systems. Goat anti-mouse podoplanin, (cat.AF3244) was purchased from Biotechne. Rabbit anti-human CD3 (cat.715903, MRQ-39) was purchased from Ionpath. Alexa 568 goat anti-mouse (cat.A21124), Alexa 568-Streptavidin (cat.S11226), and Alexa 568 goat anti-rat (cat.A11077) secondary antibodies were purchased from Invitrogen. Biotin-donkey anti-rabbit IgG (cat.3711-065-152), Cy5 donkey anti-goat IgG (cat.705-175-147) and Cy3-Streptavidin (cat.016-160-084) were purchased from Jackson. Anti-CD40 2141_IgG and 2141-V11, and DTA-1 N297A, DTA-1 IgG, DTA-1 V11 were previously reported (17).

### Plasmid constructs preparation

#### Cloning ILCD32B1

ILCD32B1 (residues 1-310) containing Y to F mutation at residue 292 in the ITIM motif was amplified by PCR using ILCD32B1pQCXIN plasmid as a template (1707, the coding sequence of ILCD32B1 was cloned between the BamHI and EcoRI restriction sites of the retroviral vector pQCXIN). NcoI and NotI recognition sites were introduced into the forward and reverse primers, respectively [For- CCTGAACCATGGTGGCCACAATGGGAATCCTGTCAT; Rev- CTATATCGCGGCCGCTGGAATTCTCAAATACGGTTCTGGTC]. The amplified DNA was digested and ligated into pBullet-EGFP (1926, the EGFP gene was cut from the pGEM4Z/GFP/A64 plasmid with NcoI and NotI and ligated into the pBullet vector) digested with the same restriction endonucleases. The resulted construct ILCD32B1pBullet, contains the ILCD32B1 gene replacing the EGFP gene in the vector. It encodes for ILCD32B1 signal peptide (1-42 AA), and the extracellular (43-217 AA), transmembrane (218-240 AA) and intracellular (241-310 AA) domains.

#### Cloning mSA2-CD8h-tILCD32B1

The sequence encoding for the monomeric streptavidin (mSA2) fused to CD8 hinge and to tILCD32B1 (residues 214-310) was ordered as dsDNA (gBlocks, IDT). Sequence was amplified by PCR and PmlI and NotI recognition sites were introduced into the forward and reverse primers, respectively [For- GTTGCCCACGTGAAGGCTGCCGCCACCATGGAGACAGACAC; Rev- CTATATCGCGGCCGCTGGAATTCTCAAATACGGTTCTGGTC]. The amplified DNA was digested and ligated into ILCD32B1pBullet digested with the same restriction endonucleases. The resulted construct mSA2-CD8h-tILCD32B1 pBullet encodes for mSA2 (including a leader sequence 20AA) fused to CD8 hinge (138-182 AA) and to tILCD32B1 (residues 214-310).

### Isolation of human peripheral blood mononuclear cells

T cells were isolated from both male and female healthy volunteer donors. The Weizmann Institute of Science Institutional Review Board, appointed by the president of the Weizmann Institute of Science, has reviewed all the experimental protocols involved in this study in accordance with the Israeli law, National Institutes of Health guidelines and the Common Rule of ethics regarding biomedical and behavioral research involving human subjects in the United States (Title 45 CFR 46). Human PBMCs were isolated by following the R&D Systems protocol for isolation of PBMCs from Whole Blood: https://www.rndsystems.com/resources/protocols/leukocyte-preparation-protocol. Human B cells were isolated by negative depletion by following the manufacturer’s instructions of the B cell isolation kit II, human (Miltenyl Biotec, cat. cat.130-091-151).

### Preparation of human IL-2 cultured effector T cells

Non-tissue culture treated 6-well plates (Falcon cat.351146) were coated with anti-CD3 (clone OKT3) and anti-CD28 (clone CD28.8) antibodies (each 1.2 mg/ml, 2.5 ml/well) overnight at 4°C. Plates were blocked with 1% BSA/PBS for 20 min. at 37°C and washed with PBS. 4 ml of human PBMCs were added to the antibody coated plates at a concentration of 1 x 10^6^/ml for 2 days. Thereafter cells were either taken for mRNA transfection or expanded every 2 days in fresh effector T cell media [RPMI (Sigma R-8758); FCS; (10%, Hyclone SH30071.03); L-Glutamine (2 mM, Sartorius 03-020-1B); Na pyruvate (1 mM, Sartorius 03-042-1B); Pen-Strep solution (Sartorius, 03-031-1B), and 2-mercaptoethanol (50 μM, Merck 805740.0250)] supplemented with IL-2 (100 units/ml, Peprotech cat.200-02). The resulting effector T cells were taken for retrovirus transduction two days after the end of their original incubation on CD3/CD28 mAb co-coated plates.

### *In vitro* transcription of mRNA

Template plasmids were linearized with SpeI. Transcription and capping reactions were carried out using T7 mScript Standard mRNA Production System (Cellscript, C-MSC11610). The mRNA product was purified by DNase-I digestion, followed by LiCl precipitation, according to the manufacturer’s instructions. The quality of the mRNA product was assessed by agarose gel electrophoresis, and concentration was determined by NanoDrop specrophotometer (Thermo Fisher Scientific, UV-VIS). Purified mRNA was stored at -80°C in small aliquots.

### mRNA electroporation of human T cells

Human effector T cells (4x10^6^, 180 ul) were electroporated with empty pulse, irrelevant, CD32B, or CD32B ITIM mutant *in vitro* transcribed mdRNA (10 ug) in Opti-MEM media in pre-chilled 2-mm cuvettes (Bio-Rad Laboratories, cat.1652086) with a Gene Pulser Xcell (Bio-Rad Laboratories) using a square-wave pulse, 380 V, 1 msec. Immediately after electroporation, cells were transferred to fresh growth medium with 3 cuvette washes with media to ensure all cells transferred.

### Transfection, preparation of packaging cell lines, and transduction of human effector T cells

The protocols used were a modified version of the methods published by Weizmann et al. (24). Tissue culture treated 6-well plates (Corning cat.3516) were coated with poly-l-lysine (0.01%, Sigma P4832, 10 min RT) and seeded with 0.5 x 10^6^ Phoenix-AMPHO (ATCC CRL-3213) cells overnight. The next morning, cells were transfected with the plasmid of interest by Lipofectamine 2000 (Invitrogen 11-668-019) per manufacturer’s instructions. The next afternoon, 0.1 x 10^6^ PG-13 cells (ATCC, CRL-10686) were seeded overnight. At 48 hrs and 72 hrs post-transfection, the media from the Phoenix cells was harvested, filtered (0.45 micron, Merck, Millex-HV Z35518), and supplemented with polybrene (8 ug/ml, Merck TR-1003-G) to infect the PG-13 cells, which were then expanded for 5-7 days and frozenin bulk. Supernatant was collected from thawed infected PG-13 cells, centrifuged at 800G for 5 min, and 4 ml added to Retronectin coated non-tissue culture plates (Falcon cat.351146), previously coated with Retronectin (Takara cat.T100A/B, 12 μg/ml, 4 ml/well) overnight at 4°C, blocked with 1% BSA/PBS for 30 min. at 37°C and washed with PBS. Viral supernatant-Retronectin plates (4 ml) were centrifuged at 2,000G for 2 hours at RT. Three ml were removed from the centrifuged viral supernatant-Retronectin plates, 2-day old effector T cells (centrifuged at 600G for 5 min and resuspended at 0.5 x 10^6^ cells/ml) were added, and T cell-viral supernatant plates were centrifuged (1,000G, 10 min) and placed in the incubator. This transduction procedure was repeated for each of the following two days for a total of three rounds of transduction.

### Flow cytometry

For analysis of surface expression of various proteins, cells were labeled with fluorescent or biotinylated primary antibodies (10 µg/ml) and washed prior to appropriate Streptavidin or species-specific secondary antibodies (1:200) in fluorescence-activated cell sorting (FACS) buffer (PBS-/-, 1% BSA, 5 mM EDTA, and 0.01% sodium azide). Flow cytometry was performed on CytoFlex flow cytometer (Beckman Coulter) and analyzed using FlowJo software v.10.7.1 (Tree Star).

### Binding assays

Effector T cells expressing mSA2-CD8h-tCD32B1 construct were incubated with fluorescent proteins (Biotin-FITC, Sigma B99431, 10 mM; PE-Biotin (Jackson ImmunoResearch 025-060-116, 1:100) for 20 min at RT, washed, and resuspended and analyzed by flow cytometry as above.

### Adhesion assays

Ibidi chambers (μ-Slide VI0.4) were coated with monoclonal antibodies, recombinant mouse ICAM-1/CD54 Fc Chimera (R&D cat. 796-IC), recombinant human CD40-Fc Chimera (R&D cat. 1493-CD), or human serum albumin (HSA), all at 10 ug/ml in PBS or coating buffer (20 mM NaHCO_3_). Effector T cells were washed in 5 mM EDTA containing medium and were suspended in binding medium (Hank’s balanced-salt solution containing 2 mg/mL BSA and 10 mM HEPES, pH 7.4). Cells were allowed to settle for 3 minutes on ICAM-1 and then were subjected to initial shear stress of 1 dyn/cm^2^ followed by ten 1 dyn/cm^2^ increases of shear stress lasting 10 secs each. In the experiments on CD40, cells were allowed to settle for 3 minutes and were subjected to an initial shear stress of 1.5 dyn/cm^2^ followed by ten 5 dyn/cm^2^ increases of shear stress, each lasting 5 sec.

T cell images were acquired by videomicroscopy at a rate of one frame every 15 seconds for 10 mins using an IX83 inverted microscope (Olympus Corporation, Tokyo, Japan) equipped with UPlanFLN 20 ×/0.50 Ph1 ∞/0.17/FN 26.5 objective (Olympus Corporation, Tokyo, Japan), 49000-ET-DAPI filter set (Chroma Technology Corp., Bellows Falls, VT, USA). ORCA-Flash4.0LT camera, model: C11440-42U (Hamamatsu Photonics K.K., Hamamatsu, Japan). Temperature was maintained at 37°C throughout the assay. For analysis, effector T cells in different fields of view (over 200 cells per field) were tracked using cellSense software 1.16 (Olympus). Data from representative experiments (N=3) were expressed as the mean range of five fields of view.

### Sample preparation for super-resolution microscopy (*d*STORM)

A suspension of effector T cells (1.5 x 10^6^) was washed with 5 mM EDTA/PBS for 5 min by centrifugation at 4°C. The cells were incubated in a blocking solution (3% BSA, PBS) on ice for 10 min. For labeling of surface molecules, the cells were treated with antibodies (10 μg/mL in 1% BSA, PBS) for 30 min on ice. After washing the cells twice with PBS by centrifugation at 4°C, they were fixed with a fixation buffer [4% (wt/vol) paraformaldehyde, 0.2%% glutaraldehyde, PBS] in suspension for 2 h on ice. The fixative was washed twice with PBS by centrifugation, resuspended in PBS and kept at 4°C. These cells were then directly used for super-resolution imaging of the surface molecule(s) of interest.

### *d*STORM imaging

Glass-bottom Petri dishes (35mm glass bottom dishes, uncoated, gamma-irradiated, Matteck, P35G-0.170-14-C) were coated with poly L-Lysine (0.01%; Sigma P4832, 1 hr, RT) and washed with PBS. Labeled cell solution (5 μL) in PBS was mixed with 150 μl of imaging buffer (7 μM glucose oxidase (Sigma), 56 nM catalase (Sigma), 20 mM cysteamine (Sigma), 50 mM Tris, 10 mM NaCl, 10% glucose, pH 8) and then was placed in the Petri dish. Cells were allowed to settle on the poly L-Lysine surface for 20 min before imaging was conducted. Three-dimensional *d*STORM imaging was preformed using Vutara SR352 microscope (Bruker) based on single-molecule localization biplane technology with 1.3 NA 60x silicon oil immersion objective (Olympus) and Hamamatsu Orca Flash 4v2 camera. The lateral localization precision is of 20-30 nanometers and axial precision is of 60-70 nanometers. A Z stack of 2-3 μm in thickness with 0.1μm slices of 400 frames each was recorded with frame rate of 50 Hz using 640 nm and 568 nm excitation lasers (maximal excitation of 6 and 10 kW/cm2 respectively).

### dSTORM data analysis

All analysis and measurement steps were performed in Vutara SRX software (version 7.0.07). Collected particles were subjected to threshold value of 20 (threshold value is defined in Vutara as signal above the frame background value). Particles were localized in three dimensions based on bead calibration. If needed, drift was corrected manually. Images were exported with the following visualization parameters: point splatting view; 50-nm particle size and opacity of 0.3. Video was created by recording the rotation of the 3D image of a cell.

### Kinetics of *in vitro* T cell decoration with biotinylated mAbs

10^6^ mSA2-CD8h-tILCD32B1 expressing effector T cell were left intact or incubated with different biotinylated rat IgG mAbs (10 µg/ml) for 20 mins at RT. Cells were washed twice and taken immediately for analysis (time zero) or suspended in T cell media at 37°C for 4 or 24 hours. The amount of decorated mAb was determined by labeling with APC conjugated anti-rat antibody and flow cytometry analysis.

### Determination of mAb decoration on the surface of effector T cells *in vivo*

All animal procedures were approved by the Weizmann Institute Institutional Animal Care and Use Committee (IACUC) and were carried out in compliance with institutional guidelines. NSG female mice (8-12 weeks old) were acclimatized for 1 week prior to experimentation. The mice were injected with ICAM-1 deficient E0771 cells (1× 10^4^ cells in 25µL PBS and 25ul Matrigel (Corning cat.536234)) into the mammary fatpads and tumors were allowed to grow for 8 days. Mice were anesthetized and 2 x10^5^ mSA2-CD8h-tILCD32B1 effector human T cells decorated with biotinylated antibodies were intratumorally injected. At different time points, the mammary fat pads were digested and harvested, and single cell suspensions were prepared and double stained for human CD3 and CD45. The amount of decorated mAb on the harvested T cells was determined by labeling with APC conjugated anti-rat antibody and flow cytometry.

### Mammary fat pad tumor harvesting and processing for flow cytometry

Upon euthanasia (CO_2_ inhalation), tumors were carefully excised from the mammary fat pads and rinsed briefly in PBS to remove excess blood and debris. The harvested mammary fat pads were finely minced into small pieces (approximately 1-2 mm³) using sterile scalpel blades in a 60 mm petri dish containing digestion media on ice [Collagenase type IV (Worthington, cat.LS004188, 1.5 mg/mL) and DNAse I (Roche, cat.10104159001, 20 ug/ml) in RPMI media]. The digested tissue was incubated with gentle agitation at 37°C for 40 minutes and after enzymatic digestion was passed through a 70um cell strainer to further remove any remaining undigested tissue or clumps. The cell suspension was then centrifuged at 1,400 rpm for 5 minutes at 4°C. The supernatant was discarded, and the cell pellet was resuspended in 1 ml RBC lysis buffer (Sigma, cat.R7757) for 5 min, neutralized with 10 ml PBS and centrifuged again. Cells were resuspended in FACS buffer and passed again through a 70um cell strainer.

### Tumor tissue fixation for immunohistochemistry and immunofluorscence staining

NSG female mice were injected with ICAM-1 deficient E0771 cells (1 × 10^4^ cells in 25 µL PBS and 25 ul Matrigel (Corning, cat.536234)) into the mammary fatpads. Tumors were allowed to grow for 8 days and mice were anaesthetized for T cell injections. 2 x10^5^ mSA2-CD8h-tILCD32B1 effector human T cells decorated with either biotinylated anti-murine podoplanin or isotype control biotin labeled mAb were intratumorally injected and 4 hours later tumors were excised and immediately fixed by submersion in 70% ethanol for 24 hours at room temperature. Fixed tumor samples were dehydrated through a graded ethanol series (70%: 1 x 45 minutes, 96%: 3 x 45 minutes, and 100%: 2 x 30 minutes) followed by xylene substitute x-tra-solve solvent (Medite 41-5213-00) diluted 1:1 with absolute ethanol for 45 minutes, solvent alone 2 x 60 minutes, and then embedded in paraffin (57°C, 3 x 60 minutes). Tissue blocks were sectioned at 4 µm thickness using a microtome (Leica RM2265) and mounted onto Superfrost Plus slides (Thermo Fisher Scientific).

For H&E staining, paraffin sections were deparaffinized by incubating slides in xylene substitute x-tra-solve solvent (3 × 5 minutes), followed by rehydration through a graded ethanol series (100%: 2 x 5 minutes, 96% and 70%, each for 5 minutes) and rinsed with tap water followed by distilled water, each for 1 minute. The slides were stained with Hematoxylin (Leica, Gill II, LE-3801521E) for 5 minutes, and rinsed with tap water for 2 minutes to remove excess hematoxylin. Next slides were incubated in acid alcohol (Ethanol 70% + 1ml HCl) for 10 seconds, followed by two rinses in tap water (3 minutes each), distilled water for 1 minute, 70% ethanol for 3 minutes, and then Eosin staining (Leica, LE-3801602E) for 1 minute 20 seconds. The slides were then dehydrated through a graded ethanol series (96%: 2 x 2 minutes, 100% each 2 x 3 minutes) and finally in solvent (2 x 3 minutes).

For immunofluorescent stainings, paraffin sections were deparaffinized by incubating slides in xylene (2 × 10 minutes), followed by rehydration through a graded ethanol series (100%, 96%, and 70%, each for 10 minutes) and rinsed with PBS.

For antigen retrieval, slides were incubated in Tris-EDTA buffer (pH 9.0). Heat-induced epitope retrieval (HIER) was performed by placing the slides in a microwave for 3 minutes at high power and for 10 minutes at 20% power. After cooling to room temperature, the slides were washed in PBS. To block nonspecific binding, tissue sections were incubated with 20% normal horse serum (Vector Laboratories) and 0.1% triton in PBS for 1.5 hours in a humidity chamber at room temperature, then subjected to biotin blocking kit (Vector Laboratories, SP-2001, per manufacturer’s instructions). The sections were then incubated overnight at 4°C with primary antibodies targeting murine podoplanin or human CD3 diluted with 2% normal horse serum and 0.1% triton in PBS. Following primary antibody incubation, slides were washed in PBS and incubated with biotin-donkey anti-rabbit IgG and Cy5 donkey anti-goat IgG antibodies for 1.5 hours at room temperature. Finally, slides were washed in PBS and incubated with Cy3-Streptavidin for 1.5 hours at room temperature, followed by washes with PBS and addition of DAPI (Sigma, cat.D952) for 1 minute followed by washing with PBS. Negative control sections were incubated with an isotype-matched control antibody or with the primary antibody omitted. Immunohistochemical and IF staining were analyzed with both a fluorescent microscope (Leica, DMi8) and a slide scanner (Panoramic Scan II, 3D Histech) at magnifications of ×10, x20 and ×40.

### Quantification and statistical analysis

Analysis was performed using Prism 9.0c version. Significance was assessed by Student’s two-tailed unpaired t test to determine the significance of the difference between means of two groups. One-way ANOVA was used to compare means among three or more independent groups.

## Results

### CD32B1 is enriched on B cell microvilli

**Figure 1:**
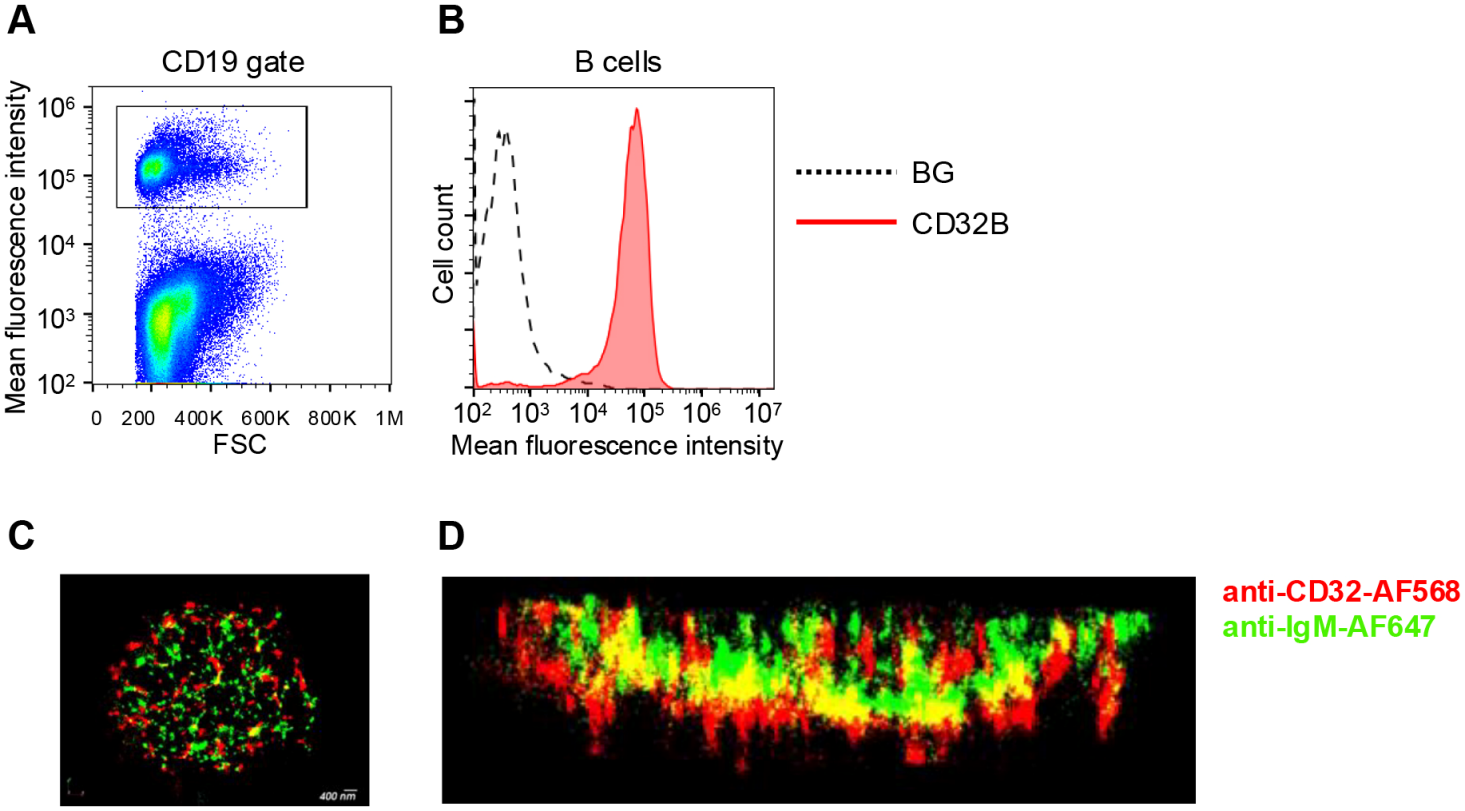
FcγRIIB (CD32B1) is expressed on tips of microvilli of B cells. **(A)** Gating strategy shown for CD19 staining of peripheral blood lymphocytes. **(B)** CD32B expression levels on the surface of B lymphocytes determined by flow cytometry (red, isotype control black dotted line). A representative experiment of three. **(C)** 3D dSTROM image of IgM (green) and CD32B (red) expression on resting B lymphocytes pre-fixed before staining in order to avoid either BCR or CD32 clustering by the staining mAbs. A representative of ten cells. **(D)** Zoomed-in and 90-rotated image of the cell.

In an attempt to choose a mAb binding scaffold that localizes to lymphocyte microvilli with high accessibility to surface bound antigens, we first assessed the distribution of native FcγRIIB1 (CD32B1) in relation to B cell microvilli. To that end, we used *d*STORM (direct stochastic optical reconstruction microscopy) super-resolution based imaging of fixed human B lymphocytes, an approach successfully used to map the distribution and proximity between different surface molecules at about 20-nm lateral and 60 nm axial resolution (25). This approach was aimed at resolving the location of this FcR nearby its main physiological target molecule, the BCR (B-cell receptor) (26). Freshly isolated B lymphocytes were purified by negative selection (**Figure 1A**) and first validated to express uniform levels of endogenous CD32B (**Figure 1B**). We expected the CD32B and IgM to colocalize, based on the postulated inhibitory role of CD32 on signaling driven by multi-valent BCR occupancy (27). Surprisingly, however, on top of substantial localization, CD32B1 was enriched within tips of microvilli whereas the BCR molecules probed by the IgM mAb appeared scattered along distinct locations within B cell MVs (**Figures 1C, D**, **Supplementary Video 1**) in agreement with recent super-resolution based studies (28, 29). This finding suggested that prior to IgM BCR occupancy by multivalent immune complexes, only a fraction of the inhibitory FcγRIIB resides in close proximity to its main target, the BCR. This result also suggested that this FcR is targeted to microvilli of B lymphocytes and can therefore potentially be used as an MV based scaffold, once introduced into other types of lymphocytes that do not express it endogenously.

### Functional ITIM-less CD32B1 is successfully expressed on the surface of effector T cells

**Figure 2:**
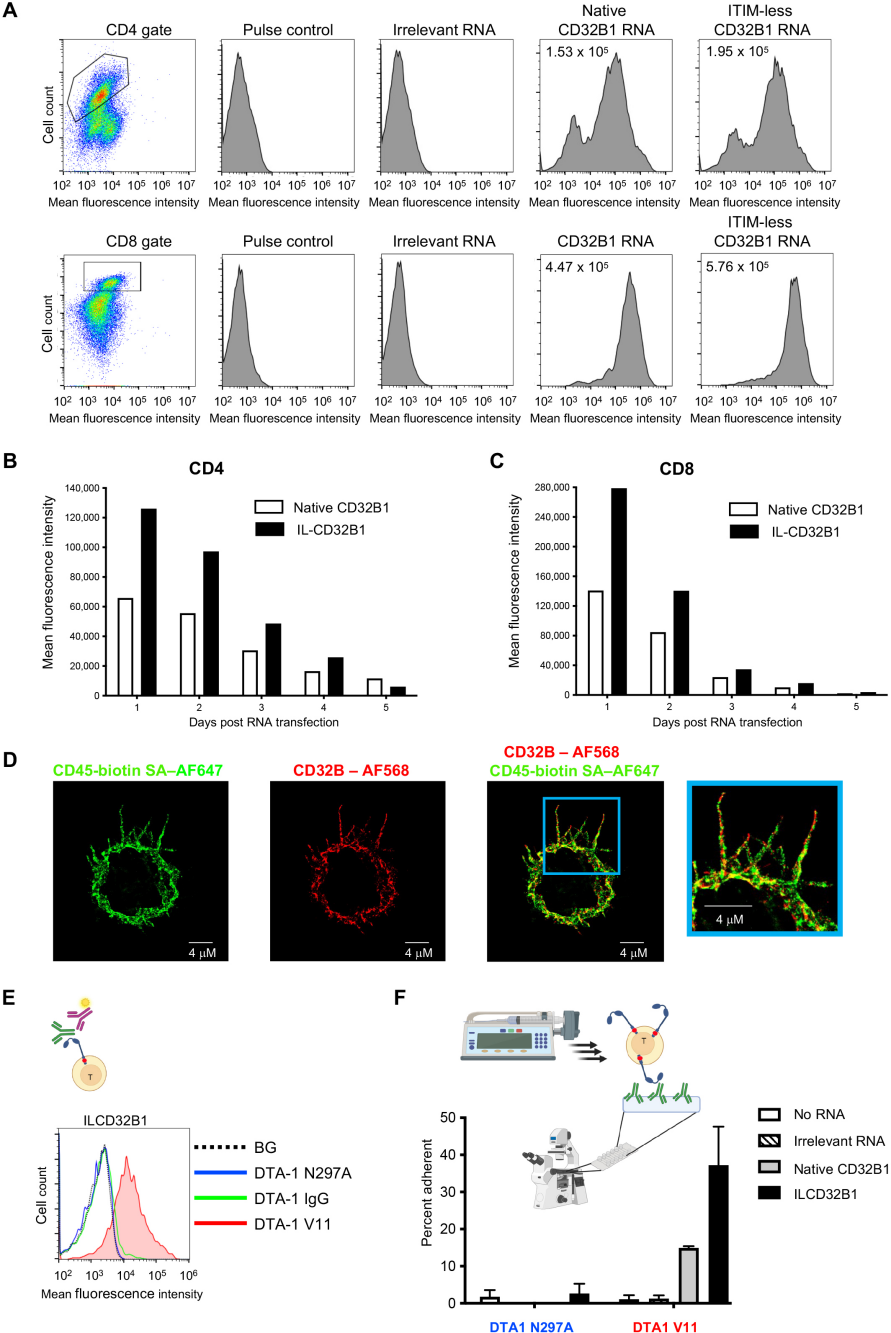
T cells expressing the ITIM-less CD32B1 bind IgG1 mutated in its Fc region and firmly adhere to a surface-bound cognate antigen. **(A)** Transient expression of native or ITIM-less FcγRIIB1 on effector CD4 (top) and CD8 (bottom) T cells. The mean fluorescence intensity is indicated in the upper left corners of representative panels. **(B, C)** Relative expression levels of either native or ITIM-less FcγRIIB1 on different times post transfection of CD4 (left) or CD8 (right) T cells with in vitro generated mRNA encoding the indicated variants. **(D)** 3D dSTROM images of effector T cells transiently expressing ITIM-less CD32B. From left to right: CD45 (green), CD32B (red), overlay of both CD32B and CD45, and zooming in the cell periphery. **(E)** Binding of the Fc V11 variant (V11, red) of the humanized anti-GITR mAb DTA-1 to T cells transiently expressing ITIM-less CD32B analyzed by fluorescent staining with secondary Ab (depicted in the graphic presentation). The ITIM domain within the cytoplasmic tail is marked as a red dot in this presentation. Binding of the N297A Fc variant that lacks any binding affinity to CD32B (N297A, blue) and of native anti-GITR IgG1 (IgG, green) to the ITIM-less CD32B expressing T cells is depicted in the respective histograms. BG- Binding of vector transfected T cells to the V11 variant. Not shown, background overlay for the native IgG and N297A variants bound to vector transfected T cells. **(F)** Adhesion of T cells transiently expressing ITIM-less CD32B1 to either the V11 Fc or the N297A Fc variants of anti-GITR mAbs each coated on a glass surface. The mAbs were coated at a density of 10 μg/ml. Shown are the fractions of T cells originally settled on the different mAb coated substrates which remained adhered and resisted detachment by high shear stresses as analyzed by videomicroscopy. Values are the mean SEM of multiple fields of view. A representative experiment of three. For more details, refer to the Materials and Methods section.

In order to assess the ability of CTLs and other effector T cells which lack endogenous CD32B expression to ectopically express either native or ITIM mutated CD32B1 on their surface, we first used an *in vitro* generated mRNA vector to introduce both the native CD32B and its ITIM-less non signaling variant into these lymphocytes (**Supplementary Figure 1**). Human T cells isolated from blood of healthy donors were *ex vivo* activated by incubation with anti CD3 and anti CD28 mAbs, transfected with the various mRNA vectors, and expanded for up to a week with IL-2. Notably, following transfection about 80% of activated CD4 and nearly all activated CD8 T cells expressed high levels of both native and ITIM-less CD32B (**Figure 2A** and **Supplementary Figure 2**). Interestingly, the relative expression levels of the ITIM-less CD32B variant on both T cell subsets were twice as high as those of the native CD32B (**Figure 2A**) consistent with a reported role of the ITIM motif in CD32B endocytosis (16). As expected, when both activated CD4 and CD8 T cells were allowed to proliferate in the IL-2 rich culture, the expression levels of both the native and ITIM-less CD32B variants on both cell subsets were reduced with each T cell division until CD32B expression was nearly completely diminished 5 days post mRNA transfection (**Figures 2B, C**).

We next followed by *d*STORM super-resolution microscopy the distribution of the ITIM-less CD32B FcR relative to T cell MV. The IL-2 cultured lymphocytes were co-incubated in the cold with fluorescently labeled antibodies to CD32B and CD45, a surface molecule uniformly distributed on T cells (28) prior to fixation. Remarkably, T cell CD32B1 was expressed on all T cell microvilli and in some lymphocytes was distributed to the T cell body (**Figure 2D**). Nevertheless, and in contrast to CD45, CD32B1 occurred in tiny patches, on T cell microvilli (**Figure 2D**, enlarged inset). To validate the functionality of the ITIM-less CD32B, with respect to IgG Fc recognition, we next assessed the ability of effector human T cells, 24 hrs post transfection with the ITIM-less CD32B-encoding mRNA to bind native IgG1. Since the Fc of IgG1 mAbs binds CD32B with moderate affinity (20, 30) we also incubated our ITIM-less CD32B expressing T cells with an Fc region variant of IgG1 termed V11 which contains an Fc insertion of 5 mutations (G237D/P238D/H268D/P271G/A330R) with 200 fold higher CD32B binding affinity compared to native IgG1 (17, 20). Accordingly, T cells expressing the ITIM-less CD32B1 efficiently bound a soluble V11 Fc mutated IgG1 mAb (**Figure 2E**) but could not bind the same mAb clone modified with a single residue mutation (N297A), reported to completely abolish CD32B binding of IgG1 Fc (**Figure 2E**). Interestingly, native soluble IgG also failed to bind T cells expressing the ITIM-less CD32B (**Figure 2E**). These results suggested that CD32B expressing T cells transduced by an mRNA encoding the FcR can be readily decorated with any IgG1 mAbs conditionally to the insertion of the V11 segment in their respective Fc region. Apart from the utility of this methodology for screens of potential CAR constructs, the introduction of an mRNA which gets diluted with T cell division seems highly useful if a transient expression of CD32B on the T cell surface is desirable.

To support efficient adhesiveness to target molecules, cell surface receptors occupied by high affinity ligands (or mAbs) must be properly anchored to the cortical actin cytoskeleton underneath their plasma membrane (31, 32). We therefore next tested the ability of the native and ITIM-less CD32B1 expressed on the surface of our effector human T cells, once occupied by a V11-Fc modified IgG1 immobilized on a solid substrate, to generate firm adhesion (**Figure 2F**, scheme). To that end, we coated a glass slide with either a V11-Fc modified mAb or the non FcR binding N297A mutated IgG mAb and allowed the native or ITIM-less CD32B1 expressing T cells to settle on each of the coated mAbs. The settled T cells were then subjected to increasing shear stresses and the fraction of T cells firmly adherent to the substrate was determined. Remarkably, about 40% of ITIM less CD32B expressing T cells firmly adhered to the coated V11 Fc modified mAb (**Figure 2F**). Consistent with its lower expression levels on T cells, the native CD32B receptor conferred a smaller fraction of cultured T cells with the ability to adhere to a substrate similarly coated with the same V11 Fc-modified mAb. As expected, either the native or the ITIM-less CD32B expressing T cells failed to adhere to the non FcR binding N297A mutated IgG mAb coated on similar glass slides (**Figure 2F**). Collectively, these results indicate that ITIM-less CD32B1 ectopically expressed by T cells provides these lymphocytes with firm adhesion upon high affinity binding to a surface-immobilized Fc modified IgG1 mAb.

### Effector T cells stably expressing ITIM-less CD32B1 get decorated with an Fc modified mAb and efficiently adhere to a surface coated cognate antigen

**Figure 3:**
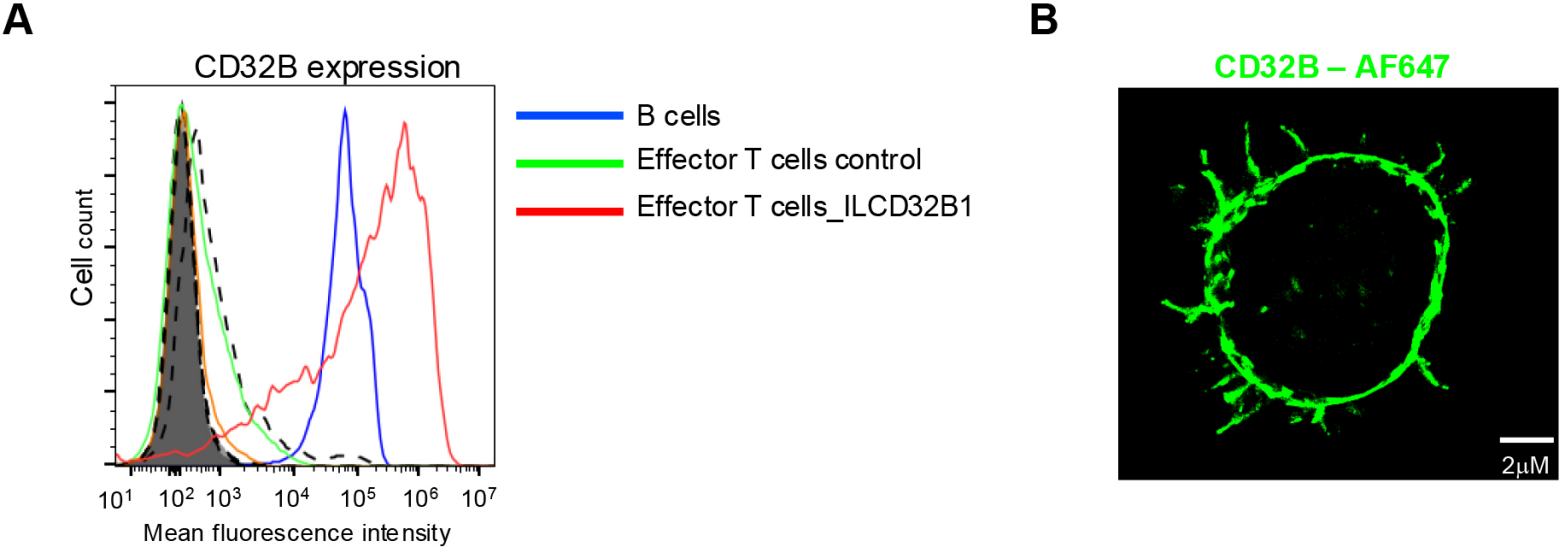
ITIM-less CD32B stably introduced into effector T cells is expressed at higher surface levels than endogenous CD32B of B lymphocytes and is distributed over the entire T cell surface. **(A)** Stable expression of ITIM-less CD32B in effector T cells introduced by retroviral mediated transduction. FACs analysis of expression levels of control or ILCD32B1 infected effector T cells determined 3 days post the third round of retroviral transduction, as compared with the expression of endogenous CD32B on the surface of B lymphocytes. Isotype control staining of ILCD32B1 infected effector T cells, control infected effector T cells, or B cells are depicted by bold dashed lines, thin dashed lines, or shaded lines, respectively. Data are displayed in a representative histogram, a representative experiment of five. **(B)** 3D dSTORM of CD32B on an effector T cell stably expressing ITIM-less CD32B. A representative cell of ten.

Having validated the functionality of the ITIM-less CD32B1 variant introduced into effector T cells, we next generated a retroviral vector encoding identical ITIM-less CD32B and introduced it into *in vitro* IL-2 cultured human effector T cells by transduction (24). This approach is extensively used for stable expression of various CARs (33, 34). Heterogenous expression of ITIM-less CD32B was obtained by this approach with a majority of effector T cells expressing 10-fold higher levels than B lymphocytes (**Figure 3A**). Importantly, these levels persisted for at least 12 days post transduction (data not shown). Interestingly, the expression levels of ITIM-less CD32B were two-fold higher than native CD32B1 (**Figure 3A**), reminiscent of the relative expression levels of the two FcR tail variants transiently introduced into effector T lymphocytes by mRNA transfection (**Figure 2**). These results further suggested that the ITIM-less CD32B mutant is intrinsically more stable than its native CD32B counterpart once transported to the plasma membrane of T cells. Similar to the effector T cells transfected with the CD32B1 encoding mRNA, the CD32B1 introduced via virus transduction was expressed on both the T cell microvilli and on the T cell body (**Figure 3B**). However, possibly due to its high density on the surface of the infected effector T cells, ITIM-less CD32B appeared uniformly distributed on the entire T cell plasma membrane.

**Figure 4:**
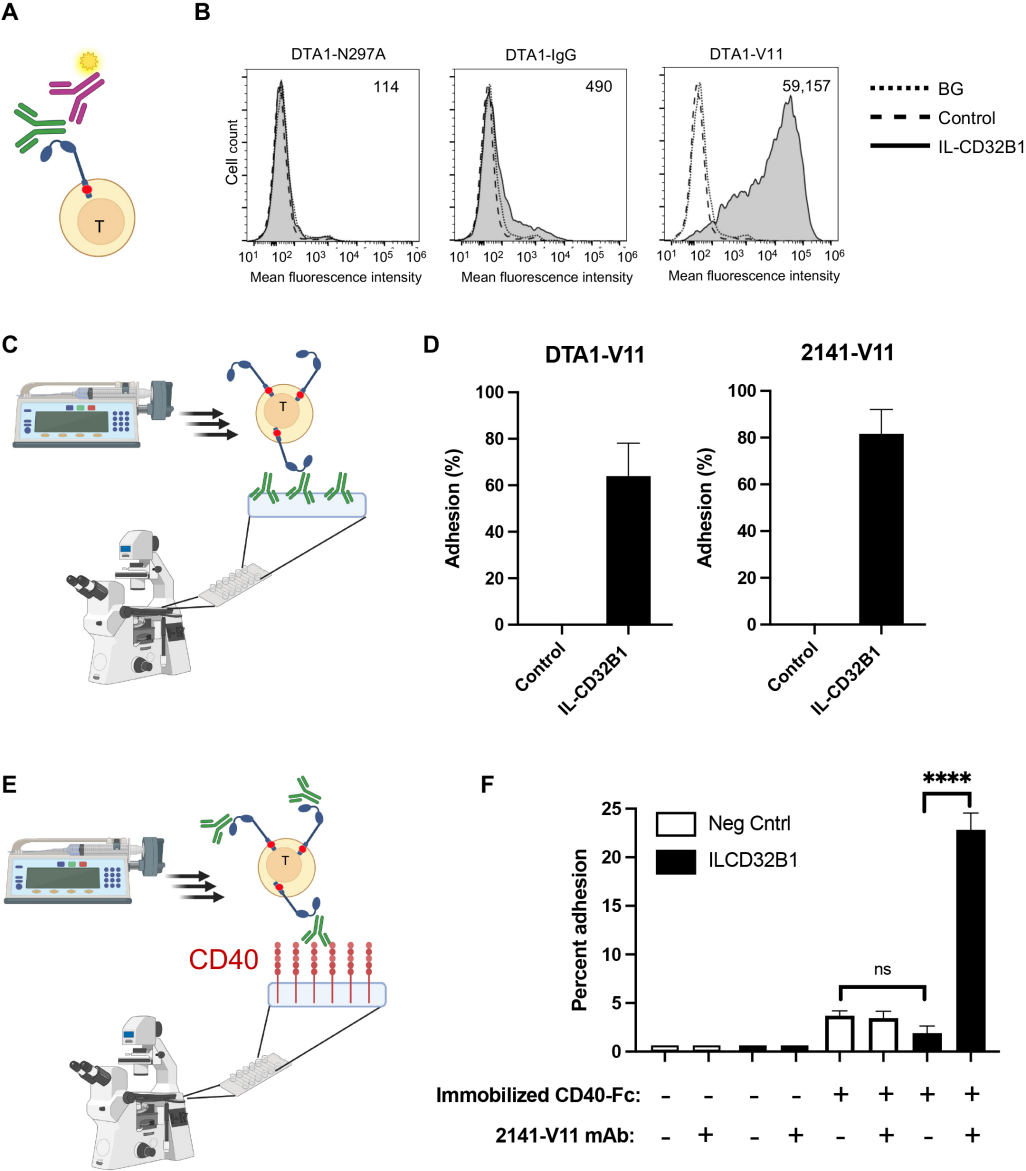
Effector T cells expressing ITIM-less CD32B efficiently bind soluble and surface coated V11-Fc modified IgG1 mAbs and stably adhere to a surface immobilized cognate antigen. **(A, B)** Binding of T cells stably expressing ITIM-less CD32B (ILCD32B1) to soluble forms of non-binding (N297A, left), native IgG (IgG, middle) and the high affinity CD32B binding V11 (V11, right) variants of humanized anti-GITR mAb DTA-1. **(A)** A scheme of the soluble antibody binding assay. Relative soluble IgG binding was determined by staining with a secondary fluorescent labeled Ab. **(B)** Dotted line corresponds to background (BG) binding of ITIM-less CD32B expressing T cells bound to isotype control; dashed line corresponds to binding of T cells infected with control vector to the indicated IgG1 mAbs; Shaded solid line: Binding of ITIM-less CD32B (ILCD32B1) expressing T cells to soluble forms of the indicated Fc variants analyzed by secondary Ab staining as explained in **(A)**. Numbers in upper right corners indicate mean fluorescent intensity values of the corresponding histograms with background staining subtracted. A representative experiment of four. **(C)** A scheme of the T cell adhesion assay. T cells are settled on substrates coated with the various Fc modified IgG1 mAbs tested in D, followed by increasing shear stresses. The fractions of the T cells originally settled on the different mAb which remained adhered and resisted detachment are determined by videomicroscopy. **(D)** Shear resistant adhesion of T cells expressing ITIM-less CD32B to surface-immobilized V11 (V11) variants of either humanized anti-GITR mAb DTA-1 or anti-CD40 mAb 2141 (2141-Vaa). Shown are the fractions of the T cells originally settled on the different mAb coated substrates which remained adhered and resisted detachment by high shear stresses. Values are the mean SEM of multiple fields of view. A representative experiment of three. **(E)** A scheme of the T cell adhesion assay. ITIM-less CD32B expressing T cells pre-decorated with V11-Fc-modified anti CD40 mAb or not, are settled on a substrate coated with the cognate antigen CD40, followed by increasing shear stresses. The fractions of the T cells which resisted detachment are determined by videomicroscopy as outlined in **(C)**. **(F)** Native relative adhesion of T cells expressing ITIM-less CD32B decorated with the V11-Fc modified anti-CD40 mAb 2141 or left intact, generated after being settled on surface-immobilized CD40. The relative shear resistant adhesion values were analyzed as in **(D)**. A representative experiment of three. ns, non-significant, p > 0.05, ****, P ≤ 0.0001

We next validated that the ITIM-less CD32B can efficiently bind the V11 modified Fc domain of two representative IgG1 mAbs both in solution and when immobilized on an adhesive substrate. As was found with T cells transiently expressing the ITIM-less CD32B (**Figure 2**), a soluble V11 Fc variant of an anti-GITR IgG mAb (DTA-1) readily bound T cells stably expressing the ITIM-less CD32B (**Figures 4A, B**). Accordingly, the vast majority of ITIM-less CD32B1 expressing T cells readily adhered to substrates coated with immobilized V11 Fc mutants of either anti GITR or anti CD40 mAbs (**Figures 4C, D**). In contrast, soluble native IgG1 anti-GITR or an N297A mutant of this IgG1 mAb failed to bind the ITIM-less CD32B expressing effector T cells (**Figure 4A**). We finally tested if an V11 Fc modified mAb occupying the CD32B1 on the surface of effector T cells can readily recognize their cognate antigens and mediated efficient T cell adhesion to a surface coated with this cognate antigen. Indeed, anti-CD40 V11 mAb-decorated CD32B1-expressing cells allowed to settle on a substrate coated with CD40 readily adhered to this antigen (**Figures 4E, F**). Importantly, the anti-CD40 mAb decorated effector T cells resisted detachment from the CD40 coated surface when subjected to high shear forces. In contrast, only a small fraction of non-decorated effector T cells adhered to the surface bound CD40, possibly via their endogenous CD40L (**Figure 4F**, bars 5-7). Thus, the CD32B-V11 Fc modified mAb complex occupied by surface-immobilized antigen confers effector T cells with stable adhesion.

### Construction of a CD32-streptavidin scaffold for surface T cell decoration with biotin labeled mAbs

**Figure 5:**
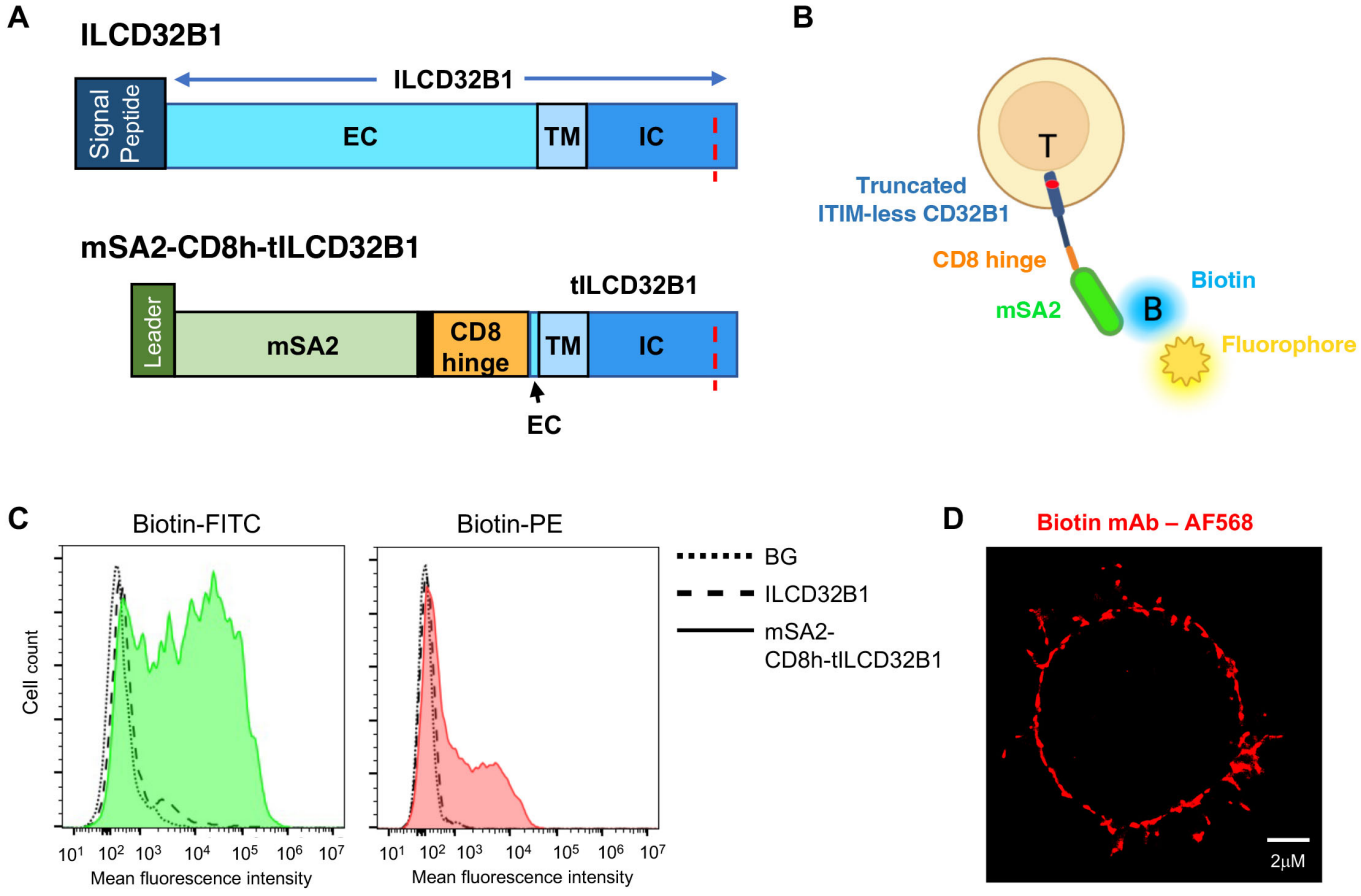
A fusion protein composed of ITIM-less CD32B1 and the streptavidin derivative mSA2 binds biotinylated fluorophores. **(A)** Schemes depicting the ILCD32B1 (upper panel) and the mSA2-CD8h-tILCD32B1 fusion (lower panel) constructs. The red dotted line depicts the Y to F point mutation annulling the functional ITIM region in the cytoplasmic tail of CD32B1. Black in the lower panel is used to depict additional short amino acid linkers in the fusion protein. Schemes are drawn to scale. See **Supplementary Figures 1** and **Supplementary Figure 4** for details on the full DNA and aa sequences of these two constructs. **(B)** A scheme of a T cell expressing the fusion molecule decorated with a soluble biotin-labeled fluorophore. **(C)** FACs staining of mSA2-CD8h-tILCD32B1 expressing T cells decorated with the indicated biotin-conjugated fluorescent probes. A representative experiment of five. **(D)** dSTORM image of a biotin-labeled mAb (αCD54) bound to T cells expressing mSA2-tCD32B. A representative cell of ten.

**Figure 6:**
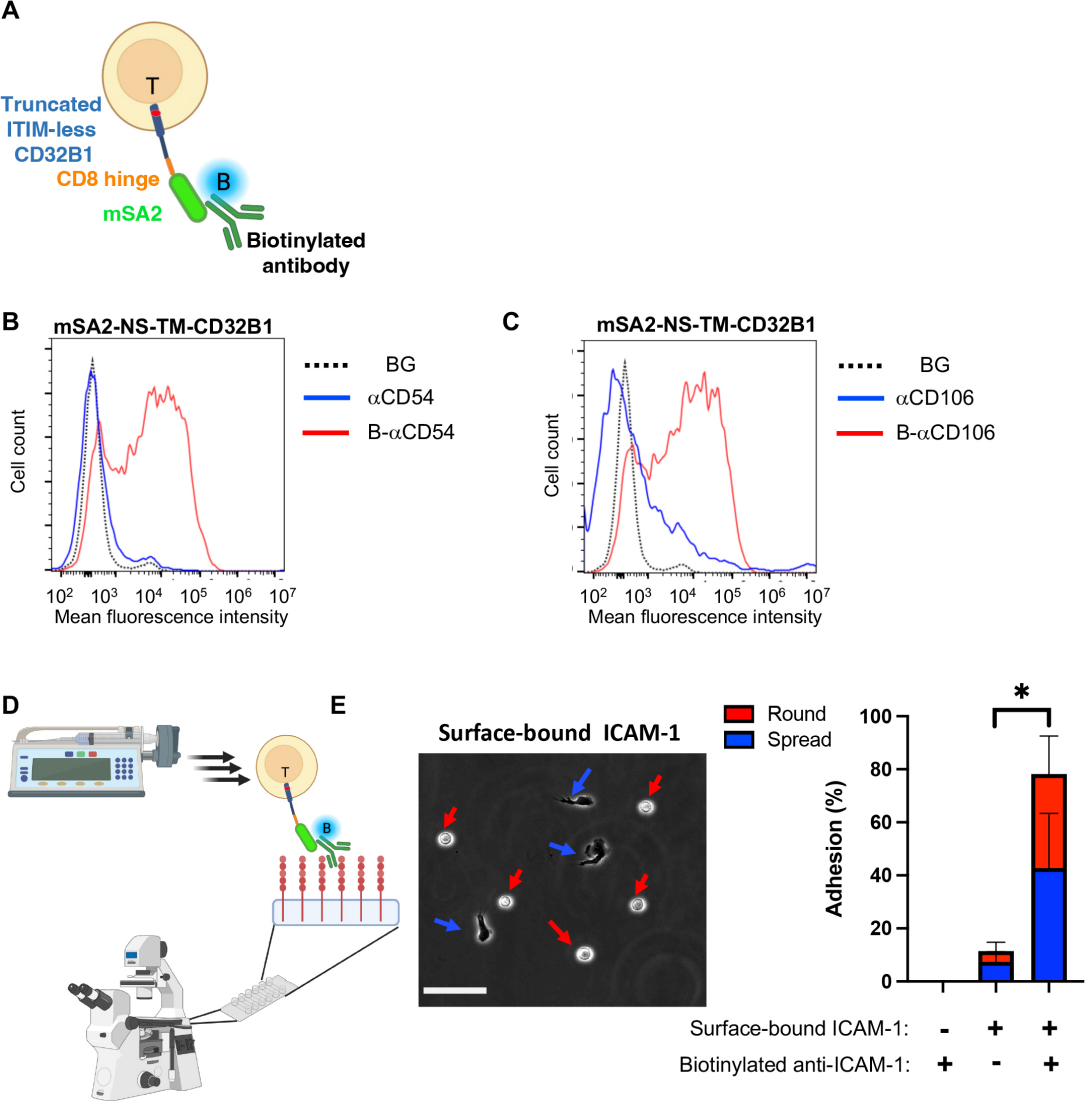
T cells expressing the CD32B streptavidin fusion protein bind biotinylated mAbs and firmly adhere to a surface-bound cognate antigen. **(A)** A scheme depicting a T cell stably expressing mSA2-CD8h-tILCD32B1 decorated with biotin labeled mAb. **(B, C)** Binding of T cells stably expressing mSA2-CD8h-tILCD32B1 to soluble polybiotinylated anti-ICAM-1 (CD54) **(B)** and anti-CD106 (VCAM-1) mAbs **(C)** analyzed by FACS. A representative experiment of five. **(D, E)** T cells expressing the mSA2-CD8h-tILCD32B1 fusion protein decorated with biotin-labeled anti-ICAM-1 readily adhere and spread on ICAM-1 coated on a glass surface (**E**, left image) and resist detachment by high shear stresses (E, right image). The same mAb decorated T cells settled on a control HSA-coated substrate were readily detached by the same shear stresses. Values are the mean SEM of multiple fields of view. A representative experiment of three conducted with binding medium supplemented with 1 mM Ca^2+^. For more details, refer to the Materials and Methods section. *, P ≤ 0.05

To bypass the need to generate individual V11-Fc modified IgG1 variants with specificity of interest, we next designed a CD32B based platform which can bind any biotin-labeled mAb of interest irrespective of its FcR recognition. To that end, we generated a fusion molecule consisting of the ITIM-less CD32B in which the entire N’ extracellular domain was substituted with a monomeric streptavidin variant termed mSA2 with intermediate affinity to biotin and biotin labeled proteins (21). The monomeric streptavidin was linked through a short hinge polypeptide derived from CD8 (23) to truncated CD32B (herein tCD32B1) followed by the entire transmembrane and the ITIM-less intracellular domains (**Figure 5A**, bottom row, and **Supplementary Figure 3**). The fusion molecule herein termed mSA2-CD8h-tCD32B1 was successfully introduced into human effector T cells with the same lentiviral vector used to introduce the ITIM-less CD32B into similar T cells (**Figure 5B**). We next validated that this fusion molecule can specifically bind both biotin-FITC and biotin-PE probes (**Figures 5C** and **Supplementary Figures 4**, **Supplementary Figure 5**). Importantly, the fusion molecule was also verified to localize to both the T cell microvilli and the T cell body (**Figure 5D**). Interestingly, the fusion molecule appeared patchy on the T cell surface reminiscent of the surface distribution of the full length CD32B1 (**Figure 2D**). Furthermore, effector T cells expressing the mSA2-CD8h-tCD32B1 fusion molecule got readily decorated with multiple biotin labeled primary mAbs as well as with a mono-biotinylated Avitag mAb fragment (**Figures 6A–C** and **Supplementary Figure 6**). Interestingly, a fusion molecule comprised of an identically truncated CD32B fused to another monomeric streptavidin variant with 7.5-fold faster biotin dissociation rate than mSA2, failed to bind biotinylated mAbs once introduced into T effectors (data not shown).

We next tested if the mSA2-tCD32B expressing T cells, once decorated with a biotin-labeled mAb with a defined specificity can readily adhere to surface-bound cognate antigen recognized by the decorating biotinylated mAb. Remarkably, when the mSA2-tCD32B expressing effector T cells decorated with a biotin labeled anti-ICAM-1 mAb were settled on a surface coated with ICAM-1, nearly all mAb decorated T cells remained stably adherent when subjected to increasing shear stresses (**Figures 6C, D**). A major fraction of the adherent lymphocytes also spread on this ligand, possibly via mAb-triggered LFA-1 outside in signaling (**Figures 6D,E**). In contrast, mSA2-tCD32B expressing T cells decorated with a biotin labeled anti-VCAM-1 failed to recognize the ICAM-1 coated substrate and readily detached from it (**Supplementary Figure 7**).

### The CD32-streptavidin scaffold promotes stable *in vitro* and *in vivo* T cell decoration with biotin labeled mAbs

**Figure 7:**
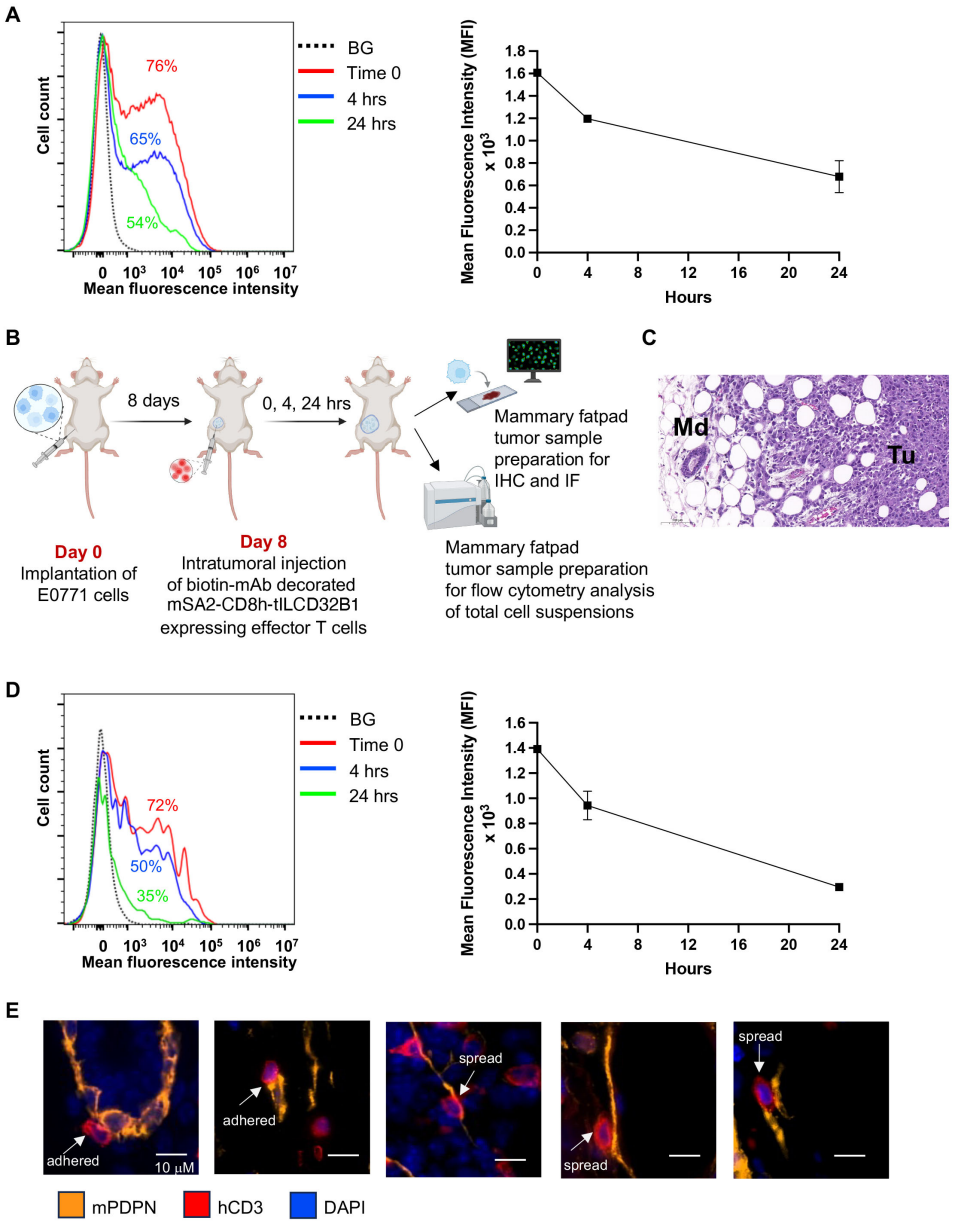
Stable in vitro and in vivo persistence of multiple mAbs on the surface of effector T cells expressing the mSA2-CD8h-tILCD32B1 scaffold. **(A)** Effector T cells were ectopically infected with mSA2-CD8h-tILCD32B1 and were either left intact or decorated with a control biotinylated rat IgG anti-B220 (not expressed in NSG mice). Cells were washed twice and taken immediately for analysis (time zero) or suspended in T cell media at 37°C for 4 or 24 hours. The amount of decorated mAb was determined by labeling with APC conjugated anti-rat antibody and flow cytometry analysis. A representative experiment of three. **(B)** A scheme depicting the experimental procedure for panels C-E. Briefly, 1x10^4^ E0771 breast cancer cells were implanted into the mammary fat pad of NSG female mice. 8 days later, dye labeled mSA2-CD8h-tILCD32B1 effector human T cells decorated with different biotin-conjugated antibodies were intratumorally injected and 0, 4 or 24 hours later, the mammary fat pads were harvested and processed for either immunohistochemistry (IHC), immunofluorescence (IF) or flow cytometry of single cell suspensions. **(C)** Hematoxylin and Eosin (H&E) staining of tumor margin area in which the location and shape of differentially decorated mSA2-CD8h-tILCD32B1 expressing effector human T cells was analyzed. Tu- tumor; Md- mammary duct. Note also the abundant white globular adipocytes in the tissue sample. A representative experiment of five. **(D)** Effector T cells expressing mSA2-CD8h-tILCD32B1 were CMTMR labeled and either left intact or decorated with a biotinylated mAb as in **(A)**. Cells were injected into mammary fatpad implanted tumors as described in **(B)**. Tumors were harvested either immediately or 4 and 24 hours later and total cell suspensions were prepared and T cell decoration with biotin labeled mAbs was analyzed by FACS as in **(A)**. A representative experiment of three. **(E)** T cells were decorated with biotin anti-podoplanin mAb and injected into mammary fatpad as described in **(B)**. Representative images for T cells (red, hCD3) adhered or spread on podoplanin expressing lymphatics (orange). DAPI staining is shown in blue. The decorated T cells did not interact with podoplanin rich CAFs (not shown). Representative images of 50.

The usage of T cell decoration with mAbs of interest would require the decorating mAb to be stably bound to the mSA2-CD8h-tILCD32B1 scaffold expressed on the surface of these lymphocytes. We therefore first determined the relative surface density of the decorated biotinylated mAb in T cells at different time points *in vitro* by FACS. As shown in **Figure 7A**, the majority of originally decorated T cells (86%) retained similar high levels of surface-bound mAb for the first 4 hours after initial mAb binding. Furthermore, by 24 hours after mAb decoration, 73% of originally decorated T cells retained the surface-bound mAb but at reduced surface density.

We next similarly assessed the relative decoration of scaffold expressing T cells *in vivo*. To this end, we established a breast cancer model based on orthotopic implantation of the breast cancer line E0771 in NSG mice (**Figures 7B, C**). The use of this immunodeficient recipient strain was aimed to minimize any potential endogenous antibody reactivity targeted at the mAb decorated human T cells studied. The mSA2-CD8h-tILCD32B1 expressing T cells were *in vitro* decorated with a biotin labeled mAb as above, and injected directly into E0771 implanted breast tumors (**Figure 7B**). Tumors were harvested either immediately or 4 and 24 hrs after injection and the relative surface density of the decorated biotinylated mAb was determined by FACS. As depicted in **Figure 7D**, the majority of originally decorated T cells (69%) retained similar levels of surface-bound mAb for the first 4 hours after initial mAb binding. Nevertheless, by 24 hours after mAb decoration, half of originally decorated T cells retained the surface-bound mAb but at diminished surface decoration densities.

Finally, we tested whether intra-tumorally injected effector T cells stably decorated with a mAb specific for a tumor enriched cell-surface marker can preferentially interact with cancer associated stromal cells expressing this marker. We chose as a model cell surface antigen podoplanin, a mucin-type transmembranal protein enriched both on tumor associated lymphatics and cancer associated fibroblasts (35, 36). Notably, the spontaneous retention of undecorated human effector T cells injected into E0771 tumor bearing mammary fatpads was much higher than in tumor-free mammary fatpads (**Supplementary Figure 9**). Nevertheless, a fraction of the tumor injected T cells decorated with biotin anti-podoplanin mAb was found to interact with tumor-associated lymphatic vessels (**Figure 7E**). Notably, close to 40% of the podoplanin-specific mAb-decorated T cells spread upon adhering to the podoplanin expressing lymphatics (**Figure 7E**). In contrast, less than 5% of the T cells which were decorated with control mAb and adhered to podoplannin expressing lymphatic cells spread on these cells.

To further assess whether the seemingly enhanced interactions between the spread mAb- decorated T cells and podoplanin rich lymphatics contribute to the overall number of T cell accumulation inside tumor bearing NSG mice recipients, we next co-injected into E0771 breast tumors equal numbers of mSA2-CD8h-tILCD32B1 expressing human T cells, differentially labeled with different cell trackers decorated with either biotinylated anti-podoplanin or isotype control biotin mAb. The overall accumulation of these two groups of effector T cells was, however, comparable both at 4 and 18 hours after intratumoral injection (**Supplementary Figure 9** and data not shown). We therefore conclude that consistent with previous reports on effector T cells accumulated in solid tumors (37), the podoplanin-specific interactions of the mAb decorated T cells were masked by high spontaneous interactions of effector lymphocytes with multiple cellular and extracellular components of the E0771 breast tumors studied by us.

## Discussion

We have applied two new genetic strategies for the decoration of effector human T cells similar to cultured CAR or TCR T cells or TILs with any mAb of interest (for a comparison of these strategies please refer to **Table 1**). The two approaches are based on an introduction of a mAb binding FcRII (CD32B) scaffold which is stably expressed on the surface of T lymphocytes that lack endogenous expression of this scaffold. The FcR contains a cytoskeletal binding cytoplasmic tail rendered non-signaling by mutation of the tail expressed ITIM motif (14–16). We have demonstrated the utility of this FcR expressed on the surface of T cells as an efficient mAb binding scaffold. Once introduced into effector T cells, this FcR could be readily occupied with both soluble and surface-immobilized IgG mAbs if these mAbs were appropriately engineered in their Fc region to bind with nM range affinities to the Fc binding site of the CD32B1. To overcome the requirement to engineer the Fc region of each mAb of interest, we have also developed a derivative of the truncated variant of the ITIM-less CD32B1, in which the extracellular IgG Fc binding domain was replaced with a monomeric avidin derivative, mSA2, which can serve as a universal scaffold for any biotin-labeled mAb (23). Importantly, these different CD32B based derivatives can each be used to decorate T cells with either a single specificity mAb or with any desirable combination of mAbs with diverse specificities. On the other hand, although a highly efficient mAb binding scaffold, the CD32-mSA2 fusion protein consists of potentially immunogenic moieties derived from bacterial streptavidin derivates (38). Repeated introduction of therapeutic CTLs decorated with such a scaffold may therefore elicit undesirable humoral responses and CTL clearance (39). On the other hand, similar fusion molecules containing streptavidin moieties have been introduced into CTLs in the past and were not reported to elicit such responses (23). Nevertheless, our tentative conclusion is that although the usage of the full length CD32B requires the Fc engineering of the mAb(s) of interest, this scaffold appears superior to the CD32-mSA2 fusion scaffold for repeated injections of CTLs into patients.

**Table 1:** A summary of the two CD32B1-based scaffolds used in the present study.

The type of scaffold	ITIM-less full CD32B1 (ILCD32B1)	Extracellular truncated ILCD32B1 fused to mSA2 (mSA2-CD8h-tILCD32B1)
The decorating mAb	Fc modified mAbs	Biotinylated mAbs
Scaffold expression level	High	High
Topographic distribution on microvilli	High	High
Reversibility of bound mAb	Variable, depending on the Fc mutation affinity	Low
Immunogenicity	Very low	Unknown
Availability	Each mAb needs to be engineered	Universal
Stability inside tumors	N.D.	Several hours

Our main goal in genetically engineering therapeutic T cells with CD32B based IgG binding scaffolds is to improve the accumulation of such T cells in solid tumors. The introduction of our different CD32-based constructs could be either stable (i.e., by infection with a retroviral vector) or transient (using mRNA introduced by electroporation). We have indeed shown that decoration of effector T cells with appropriate mAbs can confer new cell-adhesive properties to these lymphocytes, potentially facilitating their recognition of specific target molecules enriched in the tumor stroma (7). Notably, tumor associated vessels downregulate canonical vascular adhesion molecules normally recognized by therapeutic effector T cells such as the integrin ligands ICAM-1 and VCAM-1 (40–44). On the other hand, these vessels upregulate unique combinations of different cell surface proteins such as P32 (5, 8, 45, 46), Galectin-1 (9, 47), CD276 (48), Annexin-1 (10, 11, 49), CD13 (6, 12, 50, 51), and others (7). It is therefore possible that once decorated with a single mAb or with a combination of mAbs with specificities to several of these cell surface proteins, CD32B1 expressing T cells will efficiently interact with tumor associated vessels that express these multiple cell surface proteins. Likewise, surface molecules expressed by non-vascular stromal cells in the tumor microenvironment like fibroblasts and macrophages could be promising adhesive ligands for our mAb decorated effector T cells (52). In addition, extracellular matrix molecules enriched nearby cancer cells can also be attractive targets for enhanced T cell accumulation inside solid tumors (53).

Recent works suggested to utilize engineered CARs specific for tumor vascular enriched antigens in order to improve the killing efficacy of effector therapeutic T cells reaching solid tumors (54). However, this approach suffers from significant toxicity issues ranging from the destruction of healthy tissue to the cytokine release syndrome (CRS) by the CAR T cells (55). Our approach is different because it is primarily designed for introducing *de novo* adhesive properties via a non-signaling mAb-binding scaffold not designed to kill or release cytokines. To acquire these properties, we have utilized a versatile molecule endogenously localized to T cell microvilli and anchored to the cytoskeleton. We have also demonstrated its broad applicability for T cell decoration of any mAb of interest i.e., for any other stromal expressed antigen enriched the TME. The new mAb decoration approaches introduced by us can confer new cell adhesive reactivities to therapeutic T lymphocytes such as CAR or TCR T cells (CTLs) or patient derived TILs. We have also confirmed the *in vivo* stability of biotin labeled mAbs bound to our streptavidin-CD32 fusion scaffold expressed on the surface of effector T cells. We also demonstrated specific spreading of T cells decorated with anti-podoplanin on tumor associated lymphatics enriched with this surface antigen. This spreading may reflect stronger adhesive interactions between the decorated T cells and the podoplanin-rich cells. Nevertheless, in this single model tested, the specific interactions of the mAb decorated T cells with this chosen cognate antigen were most likely masked by the high spontaneous adhesion of effector lymphocytes to other cellular and extracellular components of the tumor. Future *in vivo* studies with other mAb-decorated T cells interacting with other tumors are required to assess the potential of this approach for improved migration, accumulation or killing activities of mAb decorated T cells. Such studies can open up new opportunities for new *in vivo* targeting of therapeutic T cells into different aggressive tumors.

## Data Availability

The original contributions presented in the study are included in the article/**Supplementary Material.** Further inquiries can be directed to the corresponding author.

## References

[1] Manier S Ingegnere T Escure G Prodhomme C Nudel M Mitra S. Current state and next-generation CAR-T cells in multiple myeloma. Blood Reviews. (2022) 54:100929. doi: 10.1016/j.blre.2022.100929 35131139

[2] Zhang X Zhu L Zhang H Chen S Xiao Y. CAR-T cell therapy in hematological Malignancies: current opportunities and challenges. Front Immunol. (2022) 13:927153. doi: 10.3389/fimmu.2022.927153 35757715 PMC9226391

[3] Simmons GL Castaneda Puglianini O. T-cell-based cellular immunotherapy of multiple myeloma: current developments. Cancers. (2022) 14:1–19. doi: 10.3390/cancers14174249 PMC9455067 36077787

[4] Ma S Li X Wang X Cheng L Li Z Zhang C. Current progress in car-t cell therapy for solid tumors. Int J Biol Sci. (2019) 15:2548–60. doi: 10.7150/ijbs.34213 PMC6854376 31754328

[5] Fogal V Babic I Chao Y Pastorino S Mukthavaram R Jiang P. Mitochondrial p32 is upregulated in Myc expressing brain cancers and mediates glutamine addiction. Oncotarget. (2014) 6:1157–70. doi: 10.18632/oncotarget.2708 PMC4359224 25528767

[6] Domínguez JM Pérez-Chacón G Guillén MJ Munoz-Alonso MJ Somovilla-Crespo B Cibrian D. CD13 as a new tumor target for antibody-drug conjugates: Validation with the conjugate MI130110. J Hematol Oncology. (2020) 13:1–15. doi: 10.1186/s13045-020-00865-7 PMC7140356 32264921

[7] Lugano R Ramachandran M Dimberg A. Tumor angiogenesis: causes, consequences, challenges and opportunities. Cell Mol Life sciences : CMLS. (2020) 77:1745–70. doi: 10.1007/s00018-019-03351-7 PMC7190605 31690961

[8] Sinha S Singh SK Jangde N Ray R Rai V. p32 promotes melanoma progression and metastasis by targeting EMT markers, Akt/PKB pathway, and tumor microenvironment. Cell Death Disease. (2021) 12:1–12. doi: 10.1038/s41419-021-04311-5 PMC8553772 34711805

[9] Thijssen VL Hulsmans S Griffioen AW. The galectin profile of the endothelium: Altered expression and localization in activated and tumor endothelial cells. Am J Pathol. (2008) 172:545–53. doi: 10.2353/ajpath.2008.070938 PMC2312370 18202194

[10] Oh P Testa JE Borgstrom P Witkiewicz H Li Y Schnitzer JE. *In vivo* proteomic imaging analysis of caveolae reveals pumping system to penetrate solid tumors. Nat Med. (2014) 20:1062–8. doi: 10.1038/nm.3623 25129480

[11] Allen KL Cann J Zhao W Peterson N Lazzaro M Zhong H. Upregulation of annexin A1 protein expression in the intratumoral vasculature of human non–small-cell lung carcinoma and rodent tumor models. PloS ONE. (2020) 15:1–20. doi: 10.1371/journal.pone.0234268 PMC7272081 32497150

[12] Bhagwat SV Lahdenranta J Giordano R Arap W Pasqualini R Shapiro LH. CD13/APN is activated by angiogenic signals and is essential for capillary tube formation. Blood. (2001) 97:652–9. doi: 10.1182/blood.V97.3.652 PMC4470622 11157481

[13] Pavel MA Lam C Kashyap P Salehi-Nahafabadi Z Singh G Yu Y. Analysis of the cell surface expression of cytokine receptors using the surface protein biotinylation method. Methods Mol Biol. (2014) 1172:185–92. doi: 10.1007/978-1-4939-0928-5_16 24908305

[14] Anania JC Chenoweth AM Wines BD Hogarth PM. The human FcγRII (CD32) family of leukocyte FCR in health and disease. Front Immunol. (2019) 10:464. doi: 10.3389/fimmu.2019.00464 30941127 PMC6433993

[15] Brooks DG Qiu WQ Luster AD Ravetch JV. Structure and expression of human IgG (FcRII(CD32). Functional heterogeneity is encoded by the alternatively spliced products of multiple genes. J Exp Med. (1989) 170:1369–85. doi: 10.1084/jem.170.4.1369 PMC2189488 2529342

[16] Budde P Bewarder N Weinrich V Schulzeck O Frey J. Tyrosine-containing sequence motifs of the human immunoglobulin G receptors FcRIIb1 and FcRIIb2 essential for endocytosis and regulation of calcium flux in B cells. J Biol Chem. (1994) 269:30636–44. doi: 10.1016/S0021-9258(18)43861-6 7527034

[17] Dahan R Barnhart BC Li F Yamniuk AP Korman AJ Ravetch JV. Therapeutic activity of agonistic, human anti-CD40 monoclonal antibodies requires selective fcγR engagement. Cancer Cell. (2016) 29:820–31. doi: 10.1016/j.ccell.2016.05.001 PMC4975533 27265505

[18] Li F Ravetch JV. Antitumor activities of agonistic anti-TNFR antibodies require differential FcγRIIB coengagement *in vivo* . Proc Natl Acad Sci United States America. (2013) 110:19501–6. doi: 10.1073/pnas.1319502110 PMC3845179 24218606

[19] Li F Ravetch JV. A general requirement for FcγRIIB co-engagement of agonistic anti-TNFR antibodies. Cell Cycle. (2012) 11:3343–4. doi: 10.4161/cc.21842 PMC3466534 22918247

[20] Mimoto F Katada H Kadono S Igawa T Kuramochi T Muraoka M. Engineered antibody Fc variant with selectively enhanced Fc RIIb binding over both Fc RIIaR131 and Fc RIIaH131. Protein Eng Design Selection. (2013) 26:589–98. doi: 10.1093/protein/gzt022 PMC3785249 23744091

[21] Mann JK Demonte D Dundas CM Park S. Cell labeling and proximity dependent biotinylation with engineered monomeric streptavidin. Technology. (2016) 04:152–8. doi: 10.1142/S2339547816400057

[22] Lim KH Huang H Pralle A Park S. Stabl, high-affinity streptavidin monomer for protein labeling and monovalent biotin detection. Biotechnol Bioengineering. (2013) 110:57–67. doi: 10.1002/bit.v110.1 22806584

[23] Lohmueller JJ Ham JD Kvorjak M Finn OJ. mSA2 affinity-enhanced biotin-binding CAR T cells for universal tumor targeting. OncoImmunology. (2018) 7:e1368604. doi: 10.1080/2162402X.2017.1368604 PMC5739565 29296519

[24] Weizman E Cohen CJ. Engineering T-cell specificity genetically to generate anti-melanoma reactivity. In: Methods Mol Biol (Clifton N.J.). Methods Mol Biol. (2015) pp:1–9. doi: 10.1007/7651_2015_297 26786881

[25] Rust MJ Bates M Zhuang X. Sub-diffraction-limit imaging by stochastic optical reconstruction microscopy (STORM). Nat Methods. (2006) 3:793–5. doi: 10.1038/nmeth929 PMC2700296 16896339

[26] Wang J Li Z Xu L Yang H Liu W. Transmembrane domain dependent inhibitory function of FcγRIIB. Protein Cell. (2018) 9:1004–12. doi: 10.1007/s13238-018-0509-8 PMC6251803 29497990

[27] Tzeng SJ Bolland S Inabe K Kurosaki T Pierce SK. The B cell inhibitory Fc receptor triggers apoptosis by a novel c-Abl family kinase-dependent pathway. J Biol Chem. (2005) 280:35247–54. doi: 10.1074/jbc.M505308200 16115887

[28] Jung Y Wen L Altman A Ley K. CD45 pre-exclusion from the tips of T cell microvilli prior to antigen recognition. Nat Commun. (2021) 12:1–16. doi: 10.1038/s41467-021-23792-8 34162836 PMC8222282

[29] Saltukoglu D Özdemir B Holtmannspötter M Reski R Piehler J Kurre R. Plasma membrane topography governs the 3D dynamic localization of IgM B cell antigen receptor clusters. EMBO J. (2023) 42:e112030. doi: 10.15252/embj.2022112030 36594262 PMC9929642

[30] Knorr DA Dahan R Ravetch JV. Toxicity of an Fc-engineered anti-CD40 antibody is abrogated by intratumoral injection and results in durable antitumor immunity. Proc Natl Acad Sci United States America. (2018) 115:11048–53. doi: 10.1073/pnas.1810566115 PMC6205479 30297432

[31] Dwir O Kansas GS Alon R. Cytoplasmic anchorage of L-selectin controls leukocyte capture and rolling by increasing the mechanical stability of the selectin tether. J Cell Biol. (2001) 155:145–56. doi: 10.1083/jcb.200103042 PMC2150804 11581291

[32] Alon R Feigelson SW Manevich E Rose DM Schmitz J Overby DR. Alpha4beta1-dependent adhesion strengthening under mechanical strain is regulated by paxillin association with the alpha4-cytoplasmic domain. J Cell Biol. (2005) 171:1073–84. doi: 10.1083/jcb.200503155 PMC2171310 16365170

[33] Morgan RA Boyerinas B. Genetic modification of T cells. Biomedicines. (2016) 4:9. doi: 10.3390/biomedicines4020009 28536376 PMC5344249

[34] Weinstein-Marom H Gross G Levi M Brayer H Schachter J Itzhaki O. Genetic modification of tumor-infiltrating lymphocytes via retroviral transduction. Front Immunol. (2021) 11:1-11. doi: 10.3389/fimmu.2020.584148 PMC7817656 33488585

[35] Quintanilla M Montero LM Renart J Martin-Villa E. Podoplanin in inflammation and cancer. Int J Mol Sci. (2019) 20:707. doi: 10.3390/ijms20030707 30736372 PMC6386838

[36] Astarita JL Acton SE Turley SJ. Podoplanin: Emerging functions in development, the immune system, and cancer. Front Immunol. (2012) 3:31682. doi: 10.3389/fimmu.2012.00283 PMC3439854 22988448

[37] Boissonnas A Licata F Poupel L Jacquelin S Fetler L Krumeich S. CD8+ tumor-infiltrating T cells are trapped in the tumor-dendritic cell network. Neoplasia. (2013) 15:85–94. doi: 10.1593/neo.121572 23359264 PMC3556941

[38] Yumura K Ui M Doi H Hamakubo T Kodama T Tsumoto K. Mutations for decreasing the immunogenicity and maintaining the function of core streptavidin. Protein Sci. (2013) 22:213–21. doi: 10.1002/pro.v22.2 PMC3588917 23225702

[39] Khan AN Chowdhury A Karulkar A Jaiswal AK Banik A Asija S. Immunogenicity of CAR-T cell therapeutics: evidence, mechanism and mitigation. Front Immunol. (2022) 13:14. doi: 10.3389/fimmu.2022.886546 PMC9169153 35677038

[40] Lanitis E Irving M Coukos G. Targeting the tumor vasculature to enhance T cell activity. Curr Opin Immunol. (2015) 33:55–63. doi: 10.1016/j.coi.2015.01.011 25665467 PMC4896929

[41] Dirkx AEM Oude Egbrink MGA Kuijpers MJE van der Niet ST Heijnen VVT Bouma-ter Steege JCA. Tumor angiogenesis modulates leukocyte-vessel wall interactions *in Vivo* by reducing endothelial adhesion molecule expression. Cancer Res. (2003) 63:2322–9. 12727857

[42] Schmittnaegel M De Palma M. Reprogramming tumor blood vessels for enhancing immunotherapy. Trends Cancer. (2017) 3:809–12. doi: 10.1016/j.trecan.2017.10.002 29198436

[43] Melssen MM Sheybani ND Leick KM Slingluff CL Jr. Barriers to immune cell infiltration in tumors. J ImmunoTherapy Cancer. (2023) 11:1–12. doi: 10.1136/jitc-2022-006401 PMC10124321 37072352

[44] Huang H Langenkamp E Georganaki M Loskog A Fuchs PF Dieterich LC. VEGF suppresses T-lymphocyte infiltration in the tumor microenvironment through inhibition of NF-κB-induced endothelial activation. FASEB J. (2015) 29:227–38. doi: 10.1096/fj.14-250985 25361735

[45] Rousso-Noori L Mastandrea I Talmor S Waks T Levin AG Haugas M. P32-specific CAR T cells with dual antitumor and antiangiogenic therapeutic potential in gliomas. Nat Commun. (2021) 12:1–13. doi: 10.1038/s41467-021-23817-2 34127674 PMC8203650

[46] Agemy L Kotamraju VR Friedmann-Morvinski D Sharma S Sugahara KN Ruoslahti E. Proapoptotic peptide-mediated cancer therapy targeted to cell surface p32. Mol Ther. (2013) 21:2195–204. doi: 10.1038/mt.2013.191 PMC3863797 23959073

[47] Thijssen VLJL Postel R Brandwijk RJMGE Dings RPM Nesmelova I Satijn S. Galectin-1 is essential in tumor angiogenesis and is a target for antiangiogenesis therapy. Proc Natl Acad Sci United States America. (2006) 103:15975–80. doi: 10.1073/pnas.0603883103 PMC1635112 17043243

[48] Zhou WT Jin WL. B7-H3/CD276: an emerging cancer immunotherapy. Front Immunol. (2021) 12:701006. doi: 10.3389/fimmu.2021.701006 34349762 PMC8326801

[49] Oh P Li Y Yu J Durr E Krasinska KM Carver LA. Subtractive proteomic mapping of the endothelial surface in lung and solid tumours for tissue-specific therapy. Nature. (2004) 429:629–35. doi: 10.1038/nature02580 15190345

[50] Dondossola E Rangel R Guzman-Rojas L Barbu EM Hosoya H St John LS. CD13-positive bone marrow-derived myeloid cells promote angiogenesis, tumor growth, and metastasis. Proc Natl Acad Sci United States America. (2013) 110:20717–22. doi: 10.1073/pnas.1321139110 PMC3870740 24297924

[51] Bhagwat SV Petrovic N Okamoto Y Shapiro LH. The angiogenic regulator CD13/APN is a transcriptional target of Ras signaling pathways in endothelial morphogenesis. Blood. (2003) 101:1818–26. doi: 10.1182/blood-2002-05-1422 12406907

[52] Hanahan D Coussens LM. Accessories to the crime: functions of cells recruited to the tumor microenvironment. Cancer Cell. (2012) 21:309–22. doi: 10.1016/j.ccr.2012.02.022 22439926

[53] Qiao P Lu ZR. Fibronectin in the tumor microenvironment. In: Adv Exp Med Biol Springer. (2020) pp:85–96. doi: 10.1007/978-3-030-40146-7_4 32266654

[54] Akbari P Katsarou A Daghighian R van Mil LWHG Huijbers EJM Griffioen AW. Directing CAR T cells towards the tumor vasculature for the treatment of solid tumors. Biochim Biophys Acta - Rev Cancer. (2022) 1877:188701. doi: 10.1016/j.bbcan.2022.188701 35202772

[55] Santomasso B Bachier C Westin J Rezvani K Shpall EJ. The other side of CAR T-cell therapy: cytokine release syndrome, neurologic toxicity, and financial burden. Am Soc Clin Oncol Educ Book. (2019) 39:433–44. doi: 10.1200/EDBK_238691 31099694

