# Supplementary material for: CD32B1, a versatile non-signaling antibody-binding scaffold for enhanced T cell adhesion to tumor stromal cognate antigens
Source: Front Immunol. 2025 Feb 10;16:1398757. doi: 10.3389/fimmu.2025.1398757 (PMC11847833; doi:10.3389/fimmu.2025.1398757)
Supplement: Supplementary file 2 [file Presentation1.pptx]

## Slide 1
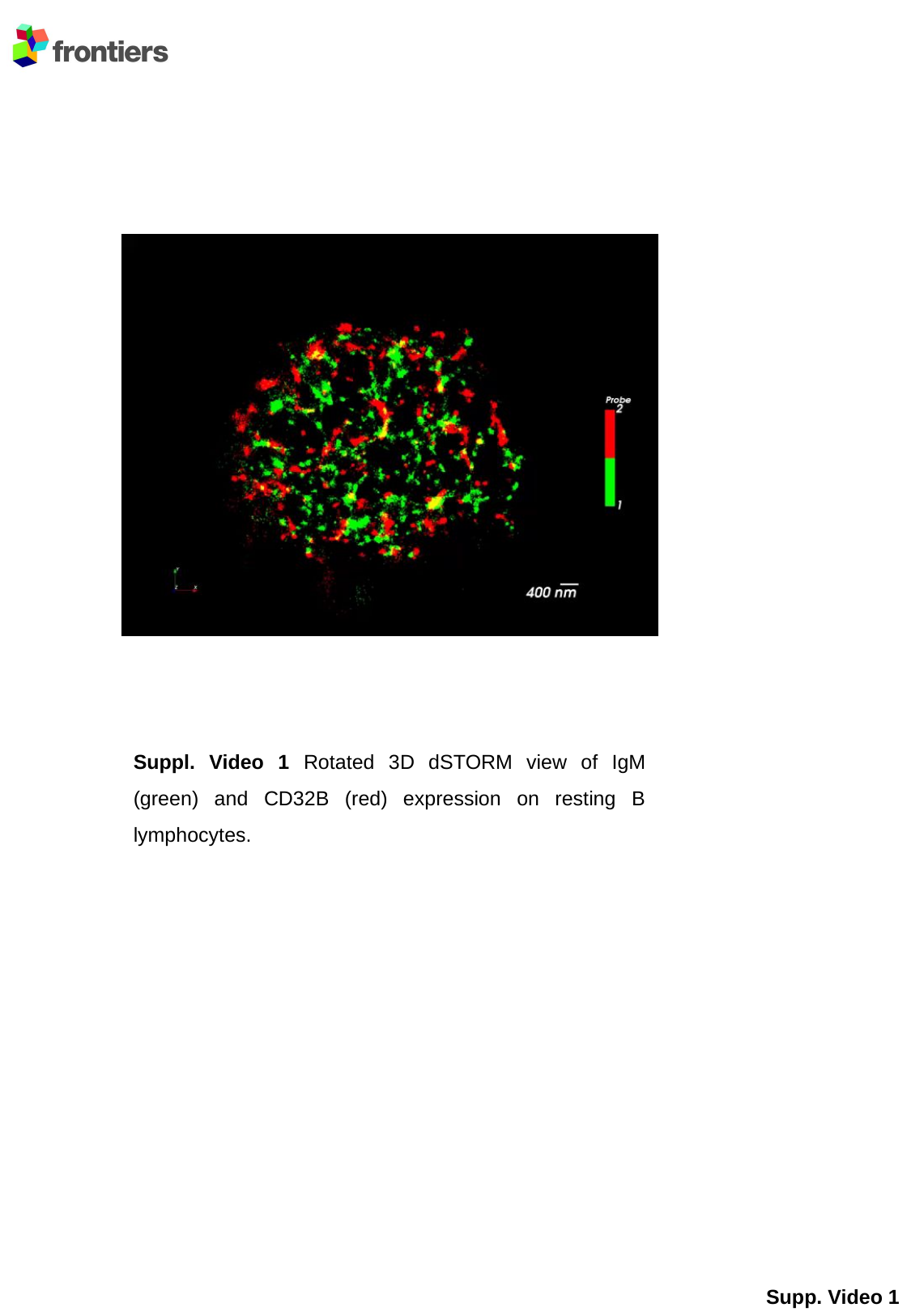

Suppl. Video 1 Rotated 3D dSTORM view of IgM (green) and CD32B (red) expression on resting B lymphocytes.
Supp. Video 1

## Slide 2
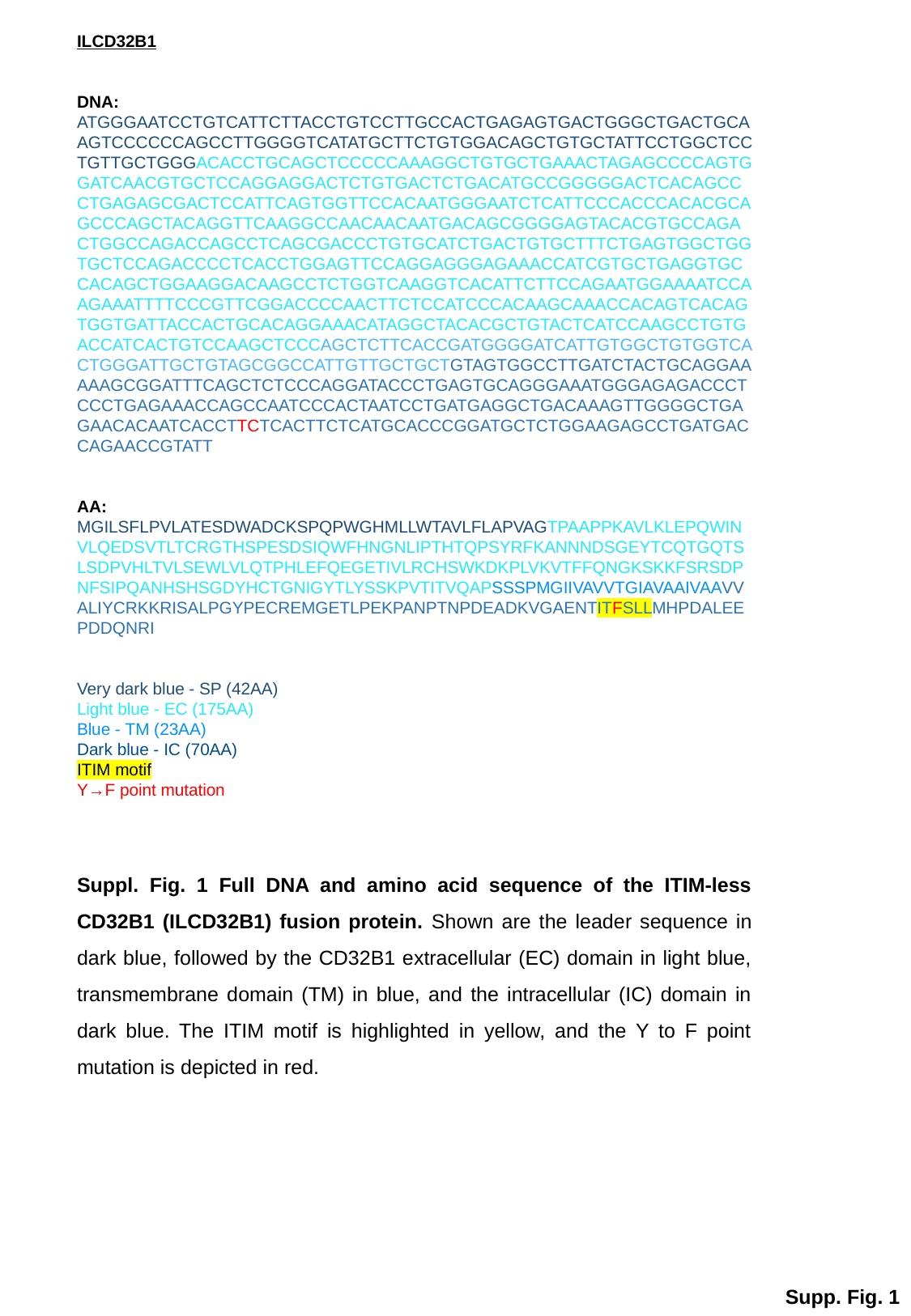

ILCD32B1
DNA:
ATGGGAATCCTGTCATTCTTACCTGTCCTTGCCACTGAGAGTGACTGGGCTGACTGCAAGTCCCCCCAGCCTTGGGGTCATATGCTTCTGTGGACAGCTGTGCTATTCCTGGCTCCTGTTGCTGGGACACCTGCAGCTCCCCCAAAGGCTGTGCTGAAACTAGAGCCCCAGTGGATCAACGTGCTCCAGGAGGACTCTGTGACTCTGACATGCCGGGGGACTCACAGCCCTGAGAGCGACTCCATTCAGTGGTTCCACAATGGGAATCTCATTCCCACCCACACGCAGCCCAGCTACAGGTTCAAGGCCAACAACAATGACAGCGGGGAGTACACGTGCCAGACTGGCCAGACCAGCCTCAGCGACCCTGTGCATCTGACTGTGCTTTCTGAGTGGCTGGTGCTCCAGACCCCTCACCTGGAGTTCCAGGAGGGAGAAACCATCGTGCTGAGGTGCCACAGCTGGAAGGACAAGCCTCTGGTCAAGGTCACATTCTTCCAGAATGGAAAATCCAAGAAATTTTCCCGTTCGGACCCCAACTTCTCCATCCCACAAGCAAACCACAGTCACAGTGGTGATTACCACTGCACAGGAAACATAGGCTACACGCTGTACTCATCCAAGCCTGTGACCATCACTGTCCAAGCTCCCAGCTCTTCACCGATGGGGATCATTGTGGCTGTGGTCACTGGGATTGCTGTAGCGGCCATTGTTGCTGCTGTAGTGGCCTTGATCTACTGCAGGAAAAAGCGGATTTCAGCTCTCCCAGGATACCCTGAGTGCAGGGAAATGGGAGAGACCCTCCCTGAGAAACCAGCCAATCCCACTAATCCTGATGAGGCTGACAAAGTTGGGGCTGAGAACACAATCACCTTCTCACTTCTCATGCACCCGGATGCTCTGGAAGAGCCTGATGACCAGAACCGTATT
AA:
MGILSFLPVLATESDWADCKSPQPWGHMLLWTAVLFLAPVAGTPAAPPKAVLKLEPQWINVLQEDSVTLTCRGTHSPESDSIQWFHNGNLIPTHTQPSYRFKANNNDSGEYTCQTGQTSLSDPVHLTVLSEWLVLQTPHLEFQEGETIVLRCHSWKDKPLVKVTFFQNGKSKKFSRSDPNFSIPQANHSHSGDYHCTGNIGYTLYSSKPVTITVQAPSSSPMGIIVAVVTGIAVAAIVAAVVALIYCRKKRISALPGYPECREMGETLPEKPANPTNPDEADKVGAENTITFSLLMHPDALEEPDDQNRI
Very dark blue - SP (42AA)
Light blue - EC (175AA)
Blue - TM (23AA)
Dark blue - IC (70AA)
ITIM motif
Y→F point mutation
Suppl. Fig. 1 Full DNA and amino acid sequence of the ITIM-less CD32B1 (ILCD32B1) fusion protein. Shown are the leader sequence in dark blue, followed by the CD32B1 extracellular (EC) domain in light blue, transmembrane domain (TM) in blue, and the intracellular (IC) domain in dark blue. The ITIM motif is highlighted in yellow, and the Y to F point mutation is depicted in red.
Supp. Fig. 1

## Slide 3
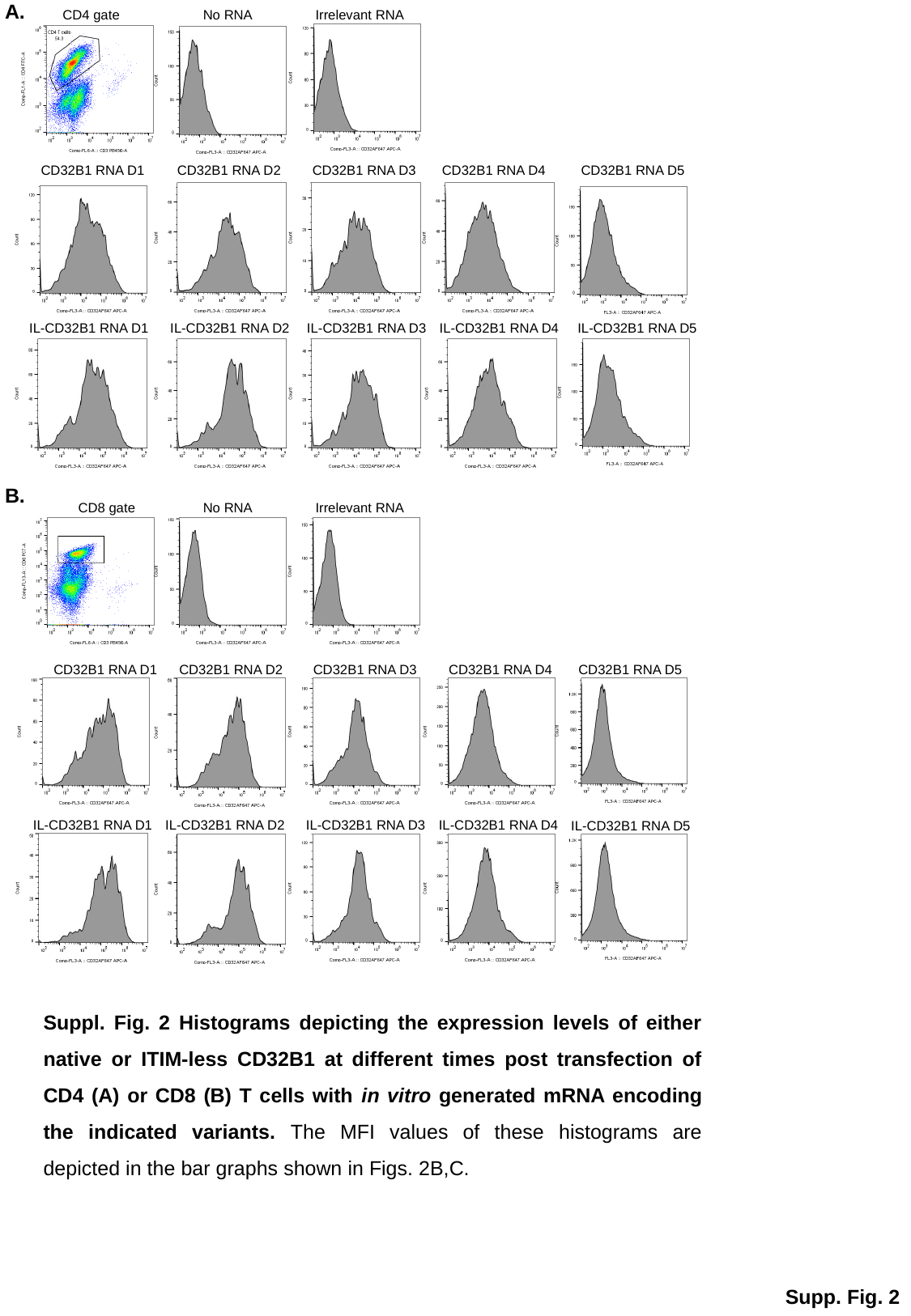

A.
CD4 gate
No RNA
Irrelevant RNA
CD32B1 RNA D4
CD32B1 RNA D1
CD32B1 RNA D2
CD32B1 RNA D3
CD32B1 RNA D5
IL-CD32B1 RNA D1
IL-CD32B1 RNA D3
IL-CD32B1 RNA D4
IL-CD32B1 RNA D5
IL-CD32B1 RNA D2
B.
CD8 gate
No RNA
Irrelevant RNA
CD32B1 RNA D1
CD32B1 RNA D2
CD32B1 RNA D3
CD32B1 RNA D4
CD32B1 RNA D5
IL-CD32B1 RNA D1
IL-CD32B1 RNA D2
IL-CD32B1 RNA D3
IL-CD32B1 RNA D4
IL-CD32B1 RNA D5
Suppl. Fig. 2 Histograms depicting the expression levels of either native or ITIM-less CD32B1 at different times post transfection of CD4 (A) or CD8 (B) T cells with in vitro generated mRNA encoding the indicated variants. The MFI values of these histograms are depicted in the bar graphs shown in Figs. 2B,C.
Supp. Fig. 2

## Slide 4
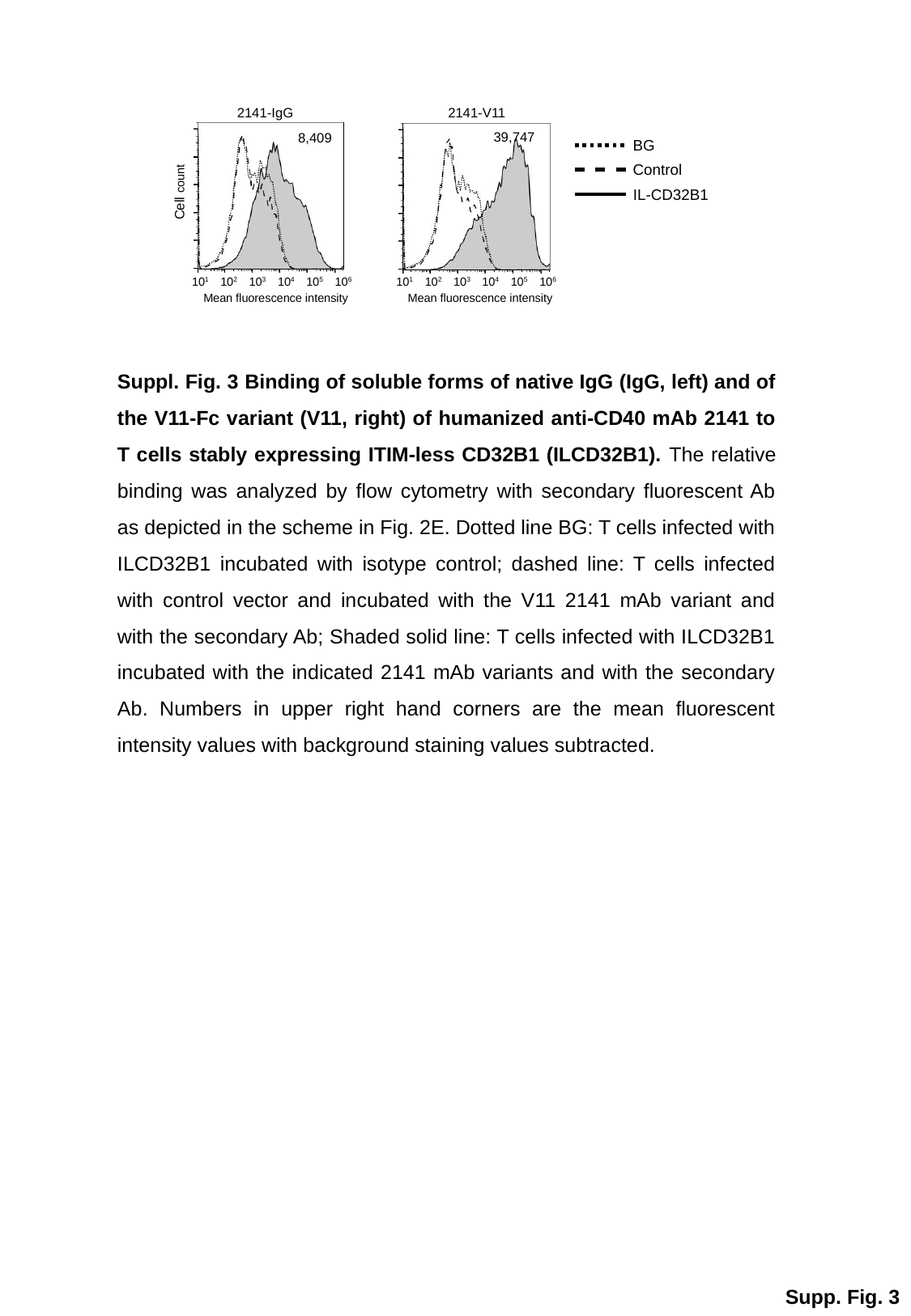

2141-IgG
2141-V11
39,747
8,409
BG
Control
IL-CD32B1
Cell count
101
102
103
104
105
106
Mean fluorescence intensity
101
102
103
104
105
106
Mean fluorescence intensity
Suppl. Fig. 3 Binding of soluble forms of native IgG (IgG, left) and of the V11-Fc variant (V11, right) of humanized anti-CD40 mAb 2141 to T cells stably expressing ITIM-less CD32B1 (ILCD32B1). The relative binding was analyzed by flow cytometry with secondary fluorescent Ab as depicted in the scheme in Fig. 2E. Dotted line BG: T cells infected with ILCD32B1 incubated with isotype control; dashed line: T cells infected with control vector and incubated with the V11 2141 mAb variant and with the secondary Ab; Shaded solid line: T cells infected with ILCD32B1 incubated with the indicated 2141 mAb variants and with the secondary Ab. Numbers in upper right hand corners are the mean fluorescent intensity values with background staining values subtracted.
Supp. Fig. 3

## Slide 5
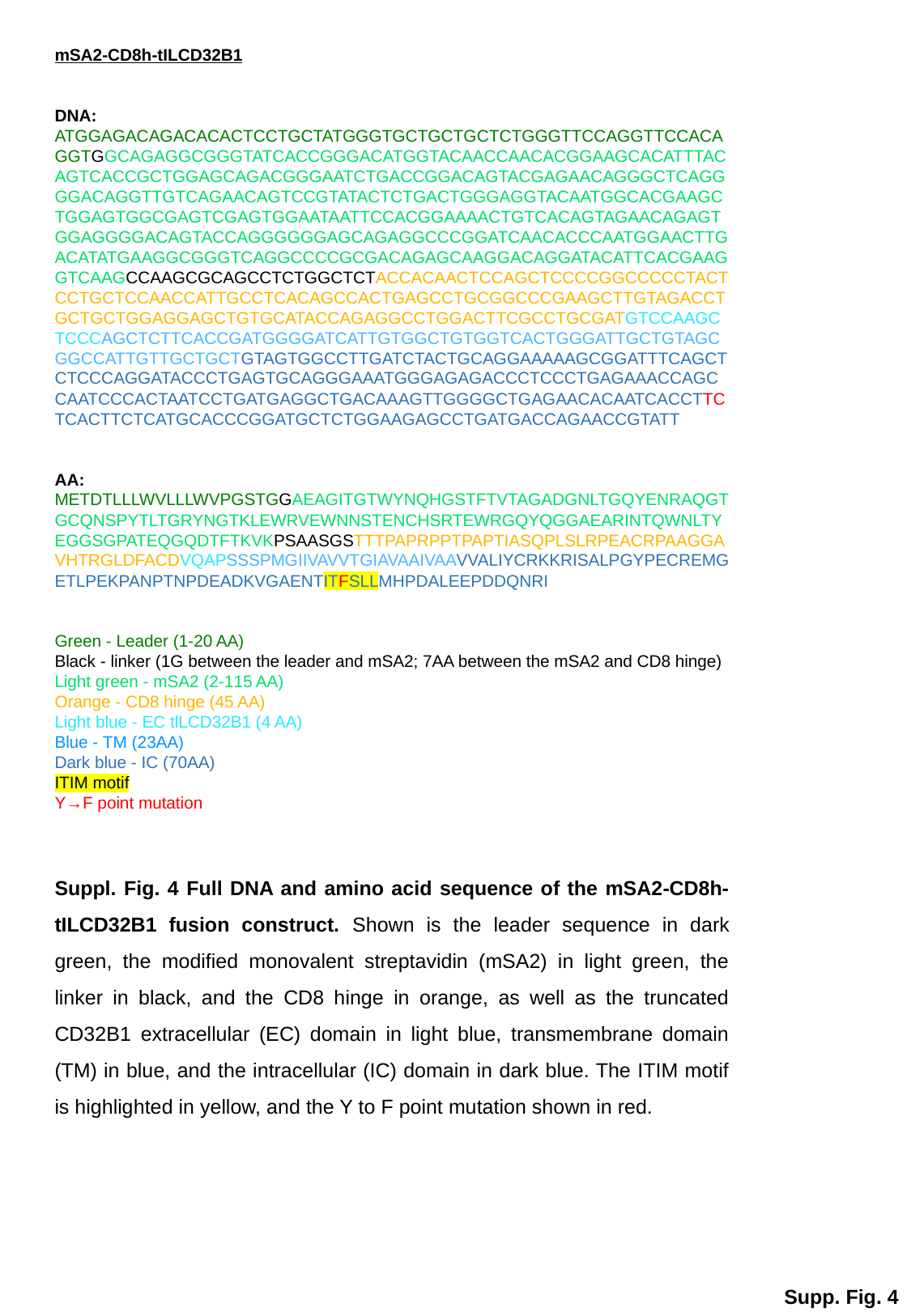

mSA2-CD8h-tILCD32B1
DNA:
ATGGAGACAGACACACTCCTGCTATGGGTGCTGCTGCTCTGGGTTCCAGGTTCCACAGGTGGCAGAGGCGGGTATCACCGGGACATGGTACAACCAACACGGAAGCACATTTACAGTCACCGCTGGAGCAGACGGGAATCTGACCGGACAGTACGAGAACAGGGCTCAGGGGACAGGTTGTCAGAACAGTCCGTATACTCTGACTGGGAGGTACAATGGCACGAAGCTGGAGTGGCGAGTCGAGTGGAATAATTCCACGGAAAACTGTCACAGTAGAACAGAGTGGAGGGGACAGTACCAGGGGGGAGCAGAGGCCCGGATCAACACCCAATGGAACTTGACATATGAAGGCGGGTCAGGCCCCGCGACAGAGCAAGGACAGGATACATTCACGAAGGTCAAGCCAAGCGCAGCCTCTGGCTCTACCACAACTCCAGCTCCCCGGCCCCCTACTCCTGCTCCAACCATTGCCTCACAGCCACTGAGCCTGCGGCCCGAAGCTTGTAGACCTGCTGCTGGAGGAGCTGTGCATACCAGAGGCCTGGACTTCGCCTGCGATGTCCAAGCTCCCAGCTCTTCACCGATGGGGATCATTGTGGCTGTGGTCACTGGGATTGCTGTAGCGGCCATTGTTGCTGCTGTAGTGGCCTTGATCTACTGCAGGAAAAAGCGGATTTCAGCTCTCCCAGGATACCCTGAGTGCAGGGAAATGGGAGAGACCCTCCCTGAGAAACCAGCCAATCCCACTAATCCTGATGAGGCTGACAAAGTTGGGGCTGAGAACACAATCACCTTCTCACTTCTCATGCACCCGGATGCTCTGGAAGAGCCTGATGACCAGAACCGTATT
AA:
METDTLLLWVLLLWVPGSTGGAEAGITGTWYNQHGSTFTVTAGADGNLTGQYENRAQGTGCQNSPYTLTGRYNGTKLEWRVEWNNSTENCHSRTEWRGQYQGGAEARINTQWNLTYEGGSGPATEQGQDTFTKVKPSAASGSTTTPAPRPPTPAPTIASQPLSLRPEACRPAAGGAVHTRGLDFACDVQAPSSSPMGIIVAVVTGIAVAAIVAAVVALIYCRKKRISALPGYPECREMGETLPEKPANPTNPDEADKVGAENTITFSLLMHPDALEEPDDQNRI
Green - Leader (1-20 AA)
Black - linker (1G between the leader and mSA2; 7AA between the mSA2 and CD8 hinge)
Light green - mSA2 (2-115 AA)
Orange - CD8 hinge (45 AA)
Light blue - EC tlLCD32B1 (4 AA)
Blue - TM (23AA)
Dark blue - IC (70AA)
ITIM motif
Y→F point mutation
Suppl. Fig. 4 Full DNA and amino acid sequence of the mSA2-CD8h-tILCD32B1 fusion construct. Shown is the leader sequence in dark green, the modified monovalent streptavidin (mSA2) in light green, the linker in black, and the CD8 hinge in orange, as well as the truncated CD32B1 extracellular (EC) domain in light blue, transmembrane domain (TM) in blue, and the intracellular (IC) domain in dark blue. The ITIM motif is highlighted in yellow, and the Y to F point mutation shown in red.
Supp. Fig. 4

## Slide 6
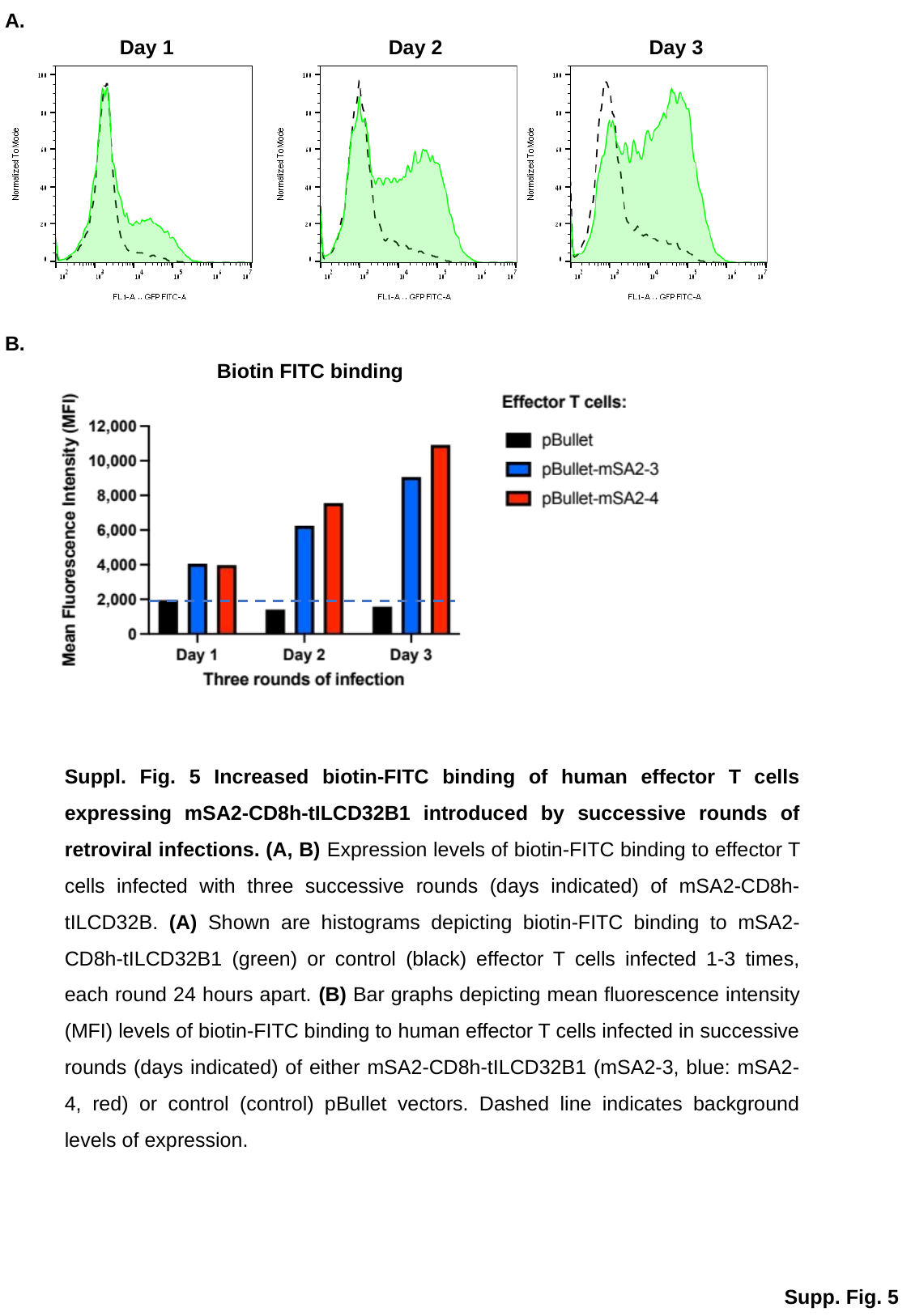

A.
Day 1
Day 2
Day 3
B.
 Biotin FITC binding
Suppl. Fig. 5 Increased biotin-FITC binding of human effector T cells expressing mSA2-CD8h-tILCD32B1 introduced by successive rounds of retroviral infections. (A, B) Expression levels of biotin-FITC binding to effector T cells infected with three successive rounds (days indicated) of mSA2-CD8h-tILCD32B. (A) Shown are histograms depicting biotin-FITC binding to mSA2-CD8h-tILCD32B1 (green) or control (black) effector T cells infected 1-3 times, each round 24 hours apart. (B) Bar graphs depicting mean fluorescence intensity (MFI) levels of biotin-FITC binding to human effector T cells infected in successive rounds (days indicated) of either mSA2-CD8h-tILCD32B1 (mSA2-3, blue: mSA2-4, red) or control (control) pBullet vectors. Dashed line indicates background levels of expression.
Supp. Fig. 5

## Slide 7
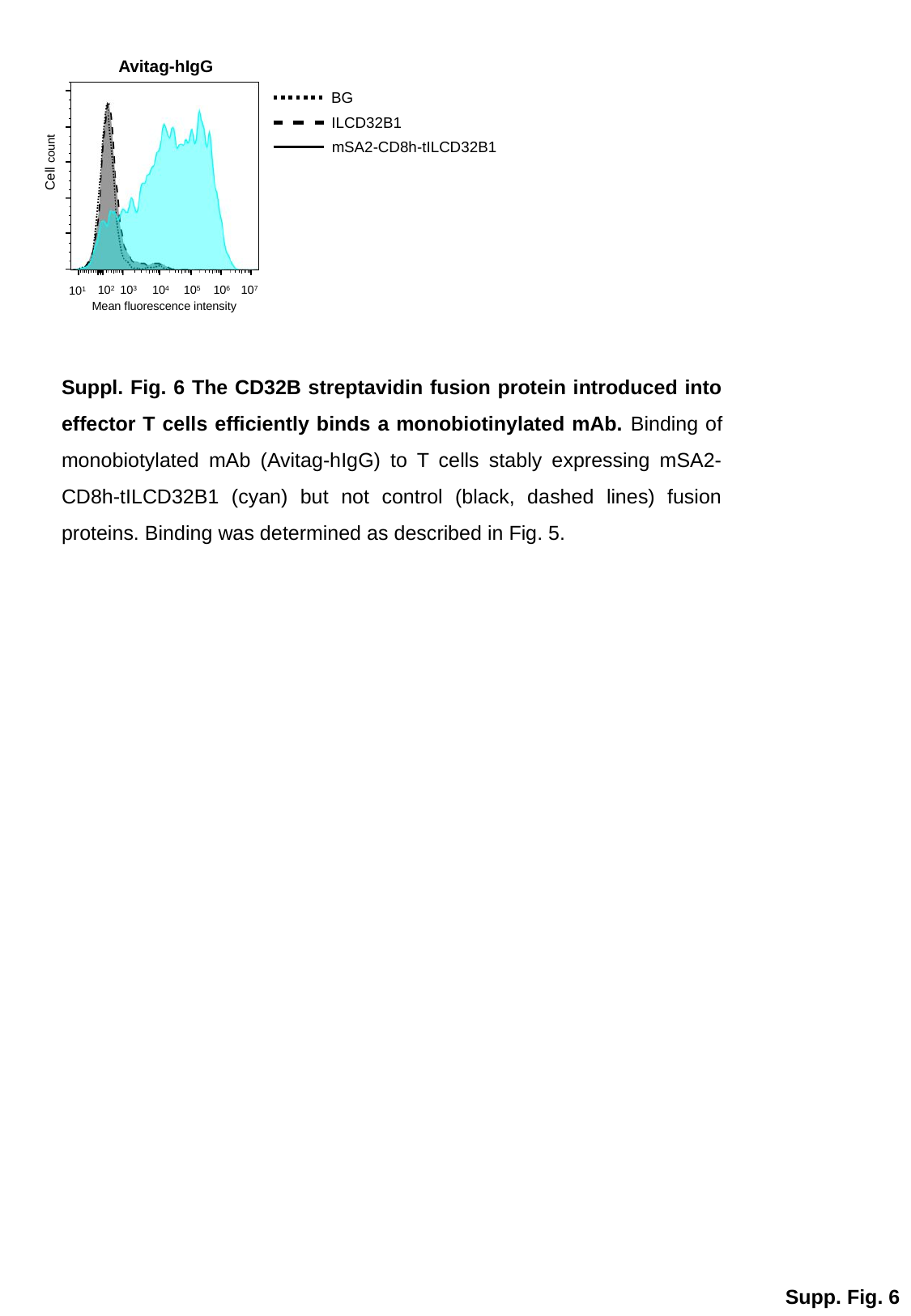

Avitag-hIgG
BG
ILCD32B1
mSA2-CD8h-tILCD32B1
Cell count
102
103
104
105
106
107
101
Mean fluorescence intensity
Suppl. Fig. 6 The CD32B streptavidin fusion protein introduced into effector T cells efficiently binds a monobiotinylated mAb. Binding of monobiotylated mAb (Avitag-hIgG) to T cells stably expressing mSA2-CD8h-tILCD32B1 (cyan) but not control (black, dashed lines) fusion proteins. Binding was determined as described in Fig. 5.
Supp. Fig. 6

## Slide 8
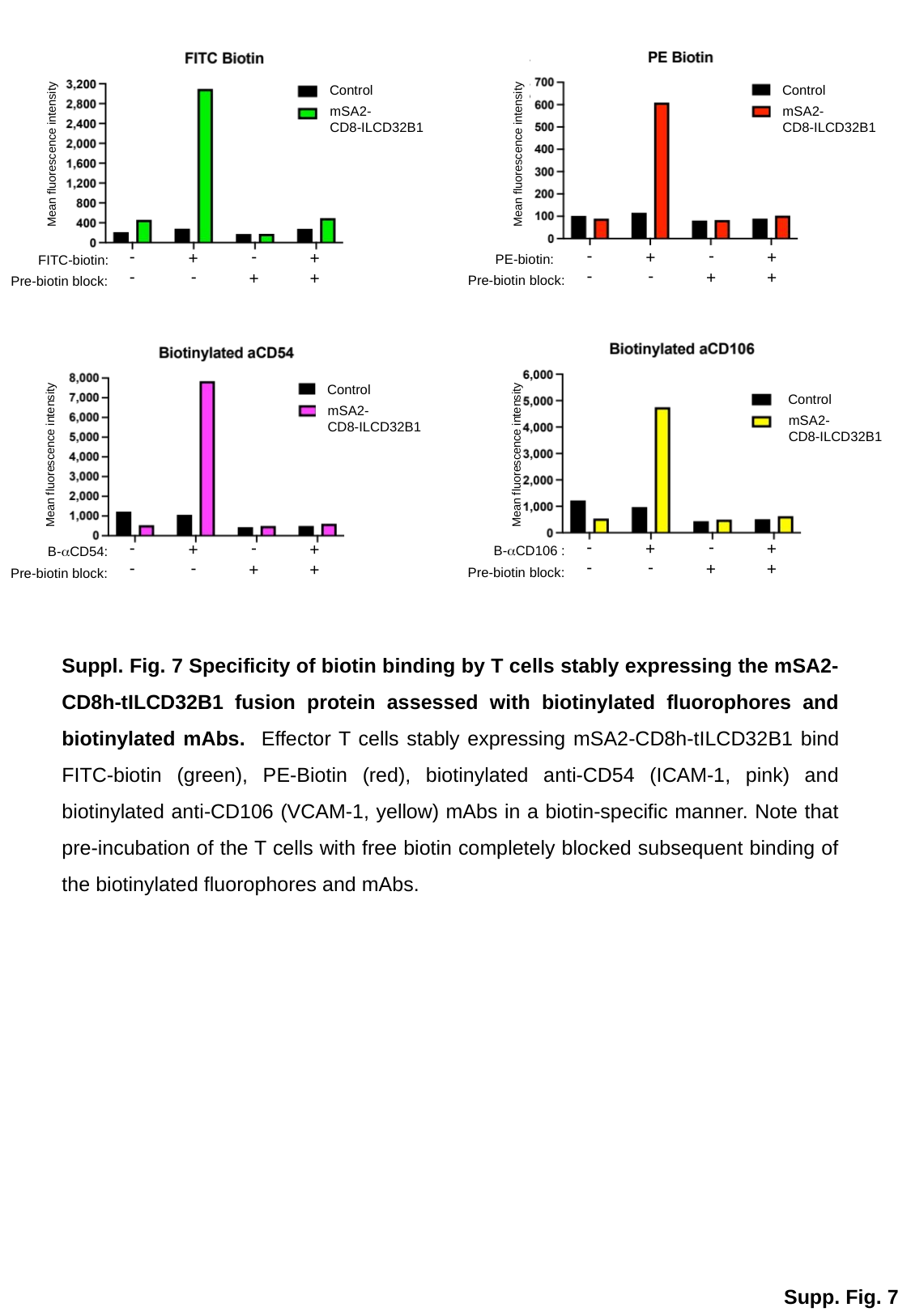

Control
mSA2-
CD8-ILCD32B1
Control
mSA2-
CD8-ILCD32B1
Mean fluorescence intensity
Mean fluorescence intensity
-
-
+
+
PE-biotin:
-
-
+
+
Pre-biotin block:
-
-
+
+
FITC-biotin:
-
-
+
+
Pre-biotin block:
Control
mSA2-
CD8-ILCD32B1
Control
mSA2-
CD8-ILCD32B1
Mean fluorescence intensity
Mean fluorescence intensity
-
-
+
+
B-aCD106 :
-
-
+
+
Pre-biotin block:
-
-
+
+
B-aCD54:
-
-
+
+
Pre-biotin block:
Suppl. Fig. 7 Specificity of biotin binding by T cells stably expressing the mSA2-CD8h-tILCD32B1 fusion protein assessed with biotinylated fluorophores and biotinylated mAbs. Effector T cells stably expressing mSA2-CD8h-tILCD32B1 bind FITC-biotin (green), PE-Biotin (red), biotinylated anti-CD54 (ICAM-1, pink) and biotinylated anti-CD106 (VCAM-1, yellow) mAbs in a biotin-specific manner. Note that pre-incubation of the T cells with free biotin completely blocked subsequent binding of the biotinylated fluorophores and mAbs.
Supp. Fig. 7

## Slide 9
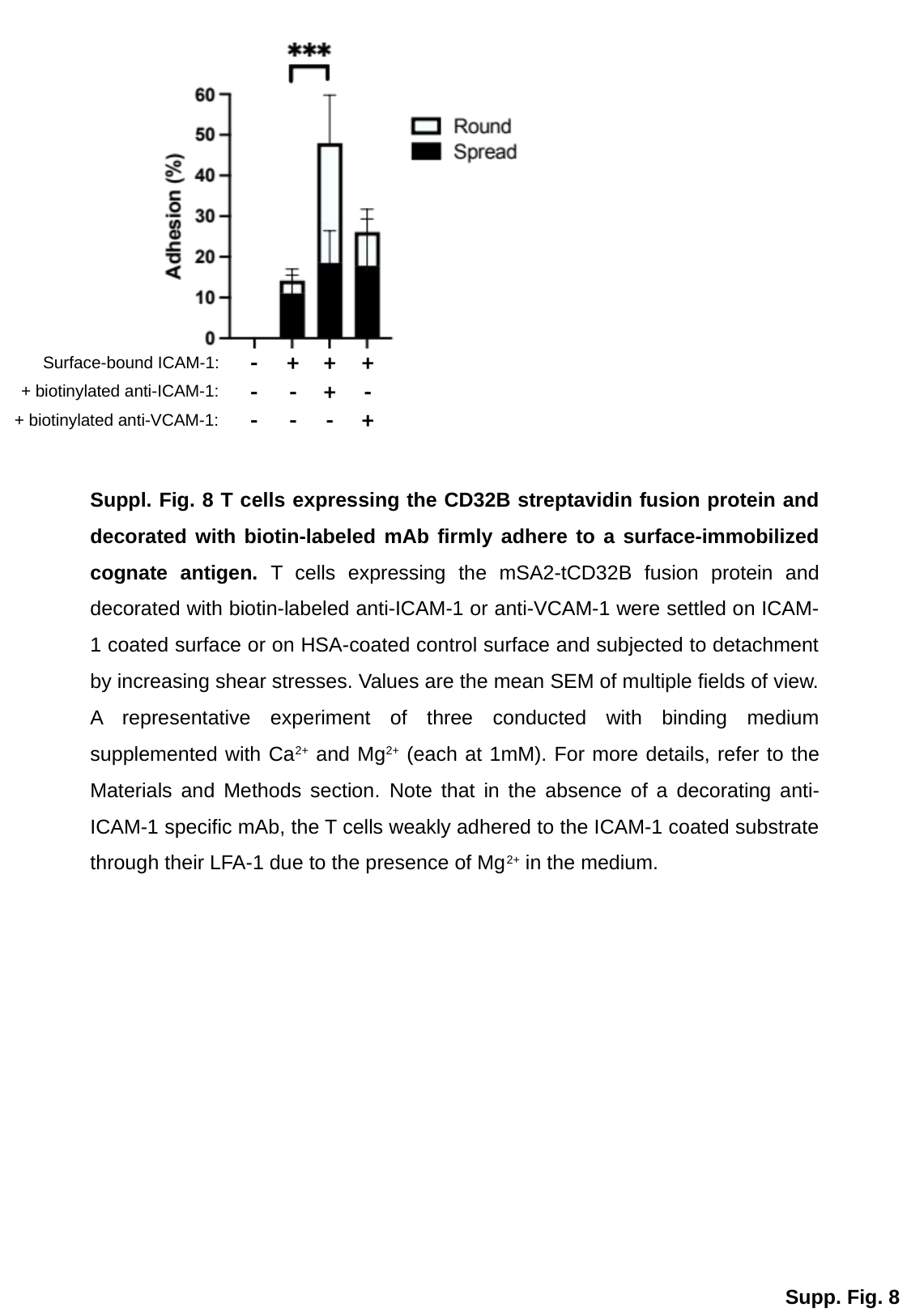

-
+
+
+
Surface-bound ICAM-1:
-
-
+
-
+ biotinylated anti-ICAM-1:
-
-
-
+
+ biotinylated anti-VCAM-1:
Suppl. Fig. 8 T cells expressing the CD32B streptavidin fusion protein and decorated with biotin-labeled mAb firmly adhere to a surface-immobilized cognate antigen. T cells expressing the mSA2-tCD32B fusion protein and decorated with biotin-labeled anti-ICAM-1 or anti-VCAM-1 were settled on ICAM-1 coated surface or on HSA-coated control surface and subjected to detachment by increasing shear stresses. Values are the mean SEM of multiple fields of view. A representative experiment of three conducted with binding medium supplemented with Ca2+ and Mg2+ (each at 1mM). For more details, refer to the Materials and Methods section. Note that in the absence of a decorating anti-ICAM-1 specific mAb, the T cells weakly adhered to the ICAM-1 coated substrate through their LFA-1 due to the presence of Mg2+ in the medium.
Supp. Fig. 8

## Slide 10
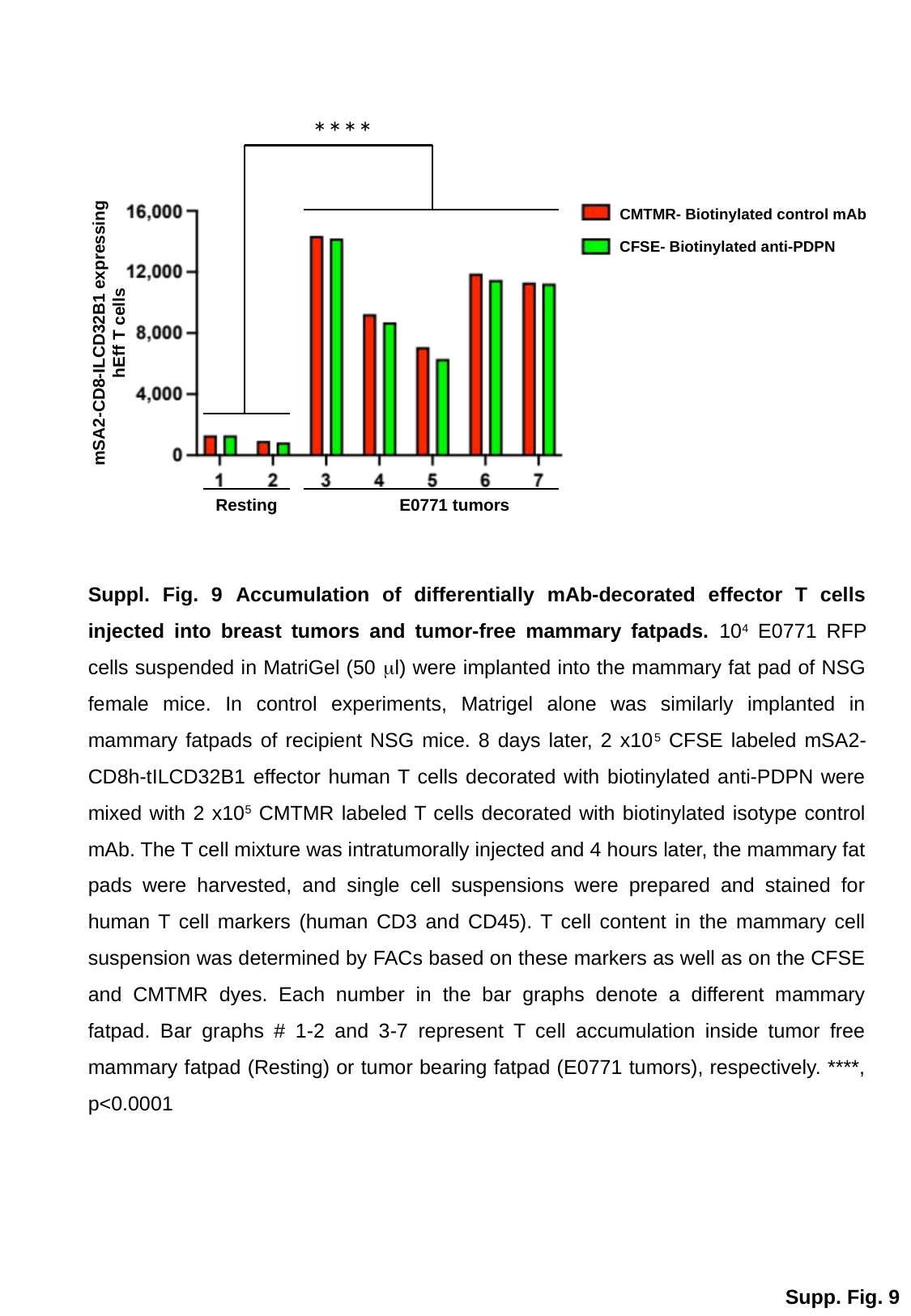

****
CMTMR- Biotinylated control mAb
CFSE- Biotinylated anti-PDPN
mSA2-CD8-ILCD32B1 expressing hEff T cells
Resting
E0771 tumors
Suppl. Fig. 9 Accumulation of differentially mAb-decorated effector T cells injected into breast tumors and tumor-free mammary fatpads. 104 E0771 RFP cells suspended in MatriGel (50 μl) were implanted into the mammary fat pad of NSG female mice. In control experiments, Matrigel alone was similarly implanted in mammary fatpads of recipient NSG mice. 8 days later, 2 x10^5^ CFSE labeled mSA2-CD8h-tILCD32B1 effector human T cells decorated with biotinylated anti-PDPN were mixed with 2 x10^5^ CMTMR labeled T cells decorated with biotinylated isotype control mAb. The T cell mixture was intratumorally injected and 4 hours later, the mammary fat pads were harvested, and single cell suspensions were prepared and stained for human T cell markers (human CD3 and CD45). T cell content in the mammary cell suspension was determined by FACs based on these markers as well as on the CFSE and CMTMR dyes. Each number in the bar graphs denote a different mammary fatpad. Bar graphs # 1-2 and 3-7 represent T cell accumulation inside tumor free mammary fatpad (Resting) or tumor bearing fatpad (E0771 tumors), respectively. ****, p<0.0001
Supp. Fig. 9
